# Ruminal Microbiota Determines the High-Fiber Utilization of Ruminants: Evidence from the Ruminal Microbiota Transplant

**DOI:** 10.1128/spectrum.00446-22

**Published:** 2022-08-04

**Authors:** Xiaodong Chen, Fang Yan, Tao Liu, Yuanling Zhang, Xinyi Li, Mengya Wang, Chenguang Zhang, Xiurong Xu, Lu Deng, Junhu Yao, Shengru Wu

**Affiliations:** a College of Animal Science and Technology, Northwest A&F University, Yangling, Shaanxi, China; b Department of Medicine, Karolinska Institute t, Solna, Stockholm, Sweden; U. S. Food and Drug Administration, National Center for Toxicological Research

**Keywords:** fiber-degrading bacteria, rumen microbe transplantation, high-fiber diet, dairy goats, mice

## Abstract

The rumen, which contains a series of prokaryotes and eukaryotes with high abundance, determines the high ability to degrade complex carbohydrates in ruminants. Using 16S rRNA gene sequencing, we compared the ruminal microbiota of dairy goats with that in the foregut and colon of mice and found more *Bacteroides* identified in the rumen, which helps ruminants to utilize plant-derived polysaccharides, cellulose, and other structural carbohydrates. Furthermore, high-fiber diets did not significantly increase intestinal fiber-degrading bacteria in mice, but did produce higher levels of ruminal fiber-degrading bacteria in dairy goats. Through rumen microbe transplantation (RMT), we found that rumen-derived fiber-degrading bacteria can colonize the intestines of mice to exert their fiber-degrading function, but their colonization efficiency is affected by diet. Additionally, the colonization of these fiber-degrading bacteria in the colon may involve higher content of butyrate in the colon, protecting the colonic epithelial barrier and promoting energy metabolism. Overall, the fiber degradation function of rumen bacteria through RMT was verified, and our results provide new insights into isolating the functional and beneficial fiber-degrading bacteria in the rumen, providing a theoretical basis for the role of dietary fiber in intestinal health.

**IMPORTANCE** Ruminants have a powerful progastric digestive system that converts structural carbohydrates into nutrients useful to humans. It is well known that this phenomenon is due to the fact that the rumen of ruminants is a natural microbial fermenter, which can ferment structural carbohydrates such as cellulose and hemicellulose and transform them into volatile fatty acids to supply energy for host. However, monogastric animals have an inherent disadvantage in utilizing fiber, so screening rumen-derived fiber-degrading bacteria as a fermentation strain for biological feed is needed in an attempt at improving the fiber digestibility of monogastric animals. In this study, a ruminal microbiota transplant experiment from goats to mice proves that ruminal microbiota could serve as a key factor in utilization of high-fiber diets and provides a new perspective for the development of probiotics with fiber degradation function from the rumen and the importance of the use of prebiotics during the intake of probiotics.

## INTRODUCTION

Nutrient utilization depends on the host’s intestinal enzymatic digestion and absorption and is affected by indirect utilization and reprocessing through the gut microbe metabolism (1–4). Expression of bacterial enzymes, such as CAZymes, could determine the utilization of nutrients and shape the intestinal microbiota (1–4). Ruminants exhibit strong abilities for fiber degradation and utilization by depending on the existence of numerous ruminal microorganisms to ferment structural carbohydrates, such as cellulose and hemicellulose, and transform them into volatile fatty acids (VFAs) absorbed by the host through the rumen wall for gluconeogenesis or by providing precursors for milk fat synthesis (5–7). Bacteria are the main components of rumen microorganisms. The *Firmicutes* and *Bacteroidetes* are the most abundant members, which contain a large number of fiber-degrading bacteria, such as Fibrobacter succinogenes, Ruminococcus flavefaciens, Ruminococcus albus, etc., which mainly degrade cellulose, and *Prevotella*, *Butyrivibrio*, *Pseudobutyrivibrio*, etc., which are known for degrading hemicellulose, and these bacteria belong to the core genera that exist in almost all ruminants (8). When these microorganisms pass through the abomasum and enter the intestines, they can be digested through host enzymes and provide a high-quality protein source for ruminants (9). Therefore, the fiber degradation process in the rumen could provide VFAs for ruminants to carry out the essential metabolic processes and promote the growth of ruminants. In contrast, fiber is always regarded as an antinutritional factor in monogastric animals. Previous studies posited that its presence would dilute the feed energy level and reduce the digestibility of nutrients, thereby affecting the animal’s performance (10, 11). This antinutritional effect is caused by the hydrophilic and highly viscous properties of soluble fiber and is related to the source of the fiber and the level of addition (12, 13). Many recent studies have begun to reveal the positive effects of dietary fiber on monogastric animal health, also through increasing the concentration of VFAs in the hindgut (14–16). We herein propose that the rumen, a natural microbial reservoir, can be used to screen rumen-derived fiber-degrading bacteria for fermentation strains for biological feed. The ruminal microbiota or some single bacteria could serve as key factors for improving the fiber digestibility of monogastric animals.

Many studies have verified the function of rumen-derived fiber-degrading microorganisms and their interactions using *in vitro* culture or coculture technology (17–19). However, the roles of rumen fiber-degrading bacteria as biological additives in promoting animal health or growth performance also need to be further explored *in vivo*, by transplanting rumen microbiota from dairy goats to mice using rumen microbe transplantation (RMT) technology. Our recent study has shown that ruminal microbiota is a key factor in the occurrence of subacute ruminal acidosis (SARA), and the RMT successfully reproduced the characteristics of goats with SARA in the colon of mice (20). Furthermore, transplanting the rumen microbes of high-yield or healthy animals to individuals with poor performance could also increase the production efficiency of the recipients (21–23). Hence, we hypothesized that RMT may also transfer such ruminant characteristics as fiber utilization ability to monogastric animals, which could prove the roles of ruminal microbiota in determining the high-fiber utilization of ruminants. Furthermore, our results also provide a new perspective for the future use of ruminal microbes to develop potential probiotics with a greater ability to degrade fiber, which can be used as a biological additive to feed that can be directly fed to animals or used in the production of fermented feed.

## RESULTS

### Comparison of the ruminal microbiota of dairy goats with the microbiota from the small intestine and colon of mice.

**FIG 1:**
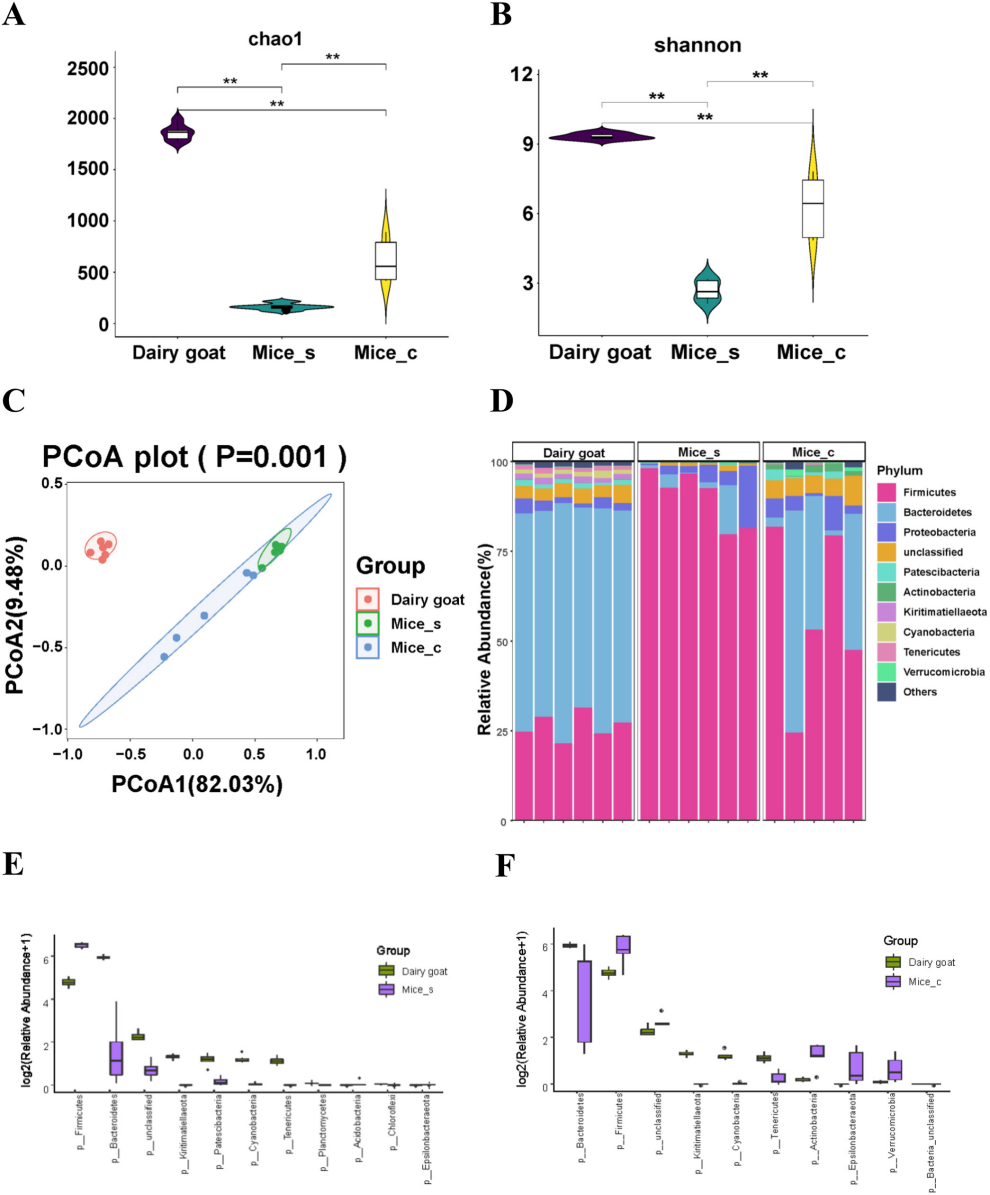
Comparison between the ruminal microbiota of dairy goats and microbiota of the small intestine and colon in mice. (A and B) Chao1 index (A) and Shannon index (B) comparison among the ruminal microbiota from goats and the small intestinal microbiota and colonic microbiomes from mice. The data were statistically analyzed using the Kruskal-Wallis test with Dunn's *post hoc* test. (C) Principal-coordinate analysis (PCoA) on rumen microbiota from dairy goats and small intestinal microbiomes and colonic microbiota from mice. The data were statistically analyzed based on ANOSIM. (D) Differences in the relative abundance of bacterial phylum levels among the rumen microbiota from goats and the small intestinal microbiota and colonic microbiota from mice; (E and F) differential bacteria at the phylum level (*P* < 0.05) between the rumen microbiota from goats and the small intestinal microbiota from mice (E) and the rumen microbiomes from goat and the colonic microbiomes from mice (F). The Mann-Whitney U test was used to identify significantly different bacteria. All bacteria listed here were significantly different, with *P* values of <0.05 between the two groups. All data are expressed as means with standard deviations.

Among all groups of dairy goats and mice, we chose dairy goats of the high-fiber diet group (HFg) as the dairy goat group, which were fed normally in production, and chose mice of the low-fiber diet (LFm) group as normal mice to compare the ruminal microbiota of dairy goats with the microbiota from the small intestine and colon of mice. The ruminal microbiota of the dairy goat group and the small intestinal and colon microbiota of mice were compared using 16S rRNA gene sequencing. The ruminal microbiota richness and diversity (Chao1 and Shannon indices) were significantly higher than those of the small intestinal and colon microbiota (Fig. 1A and B). Beta diversity analysis showed that the microbiota of dairy goats was significantly distinct from the intestinal and colonic microbiota of mice (Fig. 1C). The top 10 dominant microorganisms at the phylum level (Fig. 1D) and genus level (see Fig. S1A in the supplemental material) in the dairy goat, as well as the small intestine and colon of mice (named as Mice_s and Mice_c), were identified. At phylum levels, compared to the dairy goat, significantly higher levels of *Firmicutes*, *Acidobacteria*, and *Epsilonbacteraeota* were found in the small intestine and colon of mice, apart from higher *Verrucomicrobia* in the colon of mice, lower levels of *Bacteroidetes*, *Kiritimatiellaeota*, *Patescibacteria*, *Cyanobacteria*, *Tenericutes*, *Planctomycetes*, and *Chloroflexi* in the small intestines of mice, and significantly lower levels of *Bacteroidetes*, *Kiritimatiellaeota*, *Cyanobacteria*, and *Tenericutes* in the colon of mice (Fig. 1E and F; Table S1). Similarly, significant changes in bacteria at the genus level between the rumen of dairy goats and the small intestine and colon of mice were also identified (Fig. S1B and C and Table S2). Overall, the ruminal microbiota has more abundant fiber utilization bacteria than that identified in both the small intestinal and colonic microbiota of mice.

### The ruminal microbiota of dairy goats and intestinal microbiota of mice exhibited different alterations in response to high-fiber and low-fiber diets.

**FIG 2:**
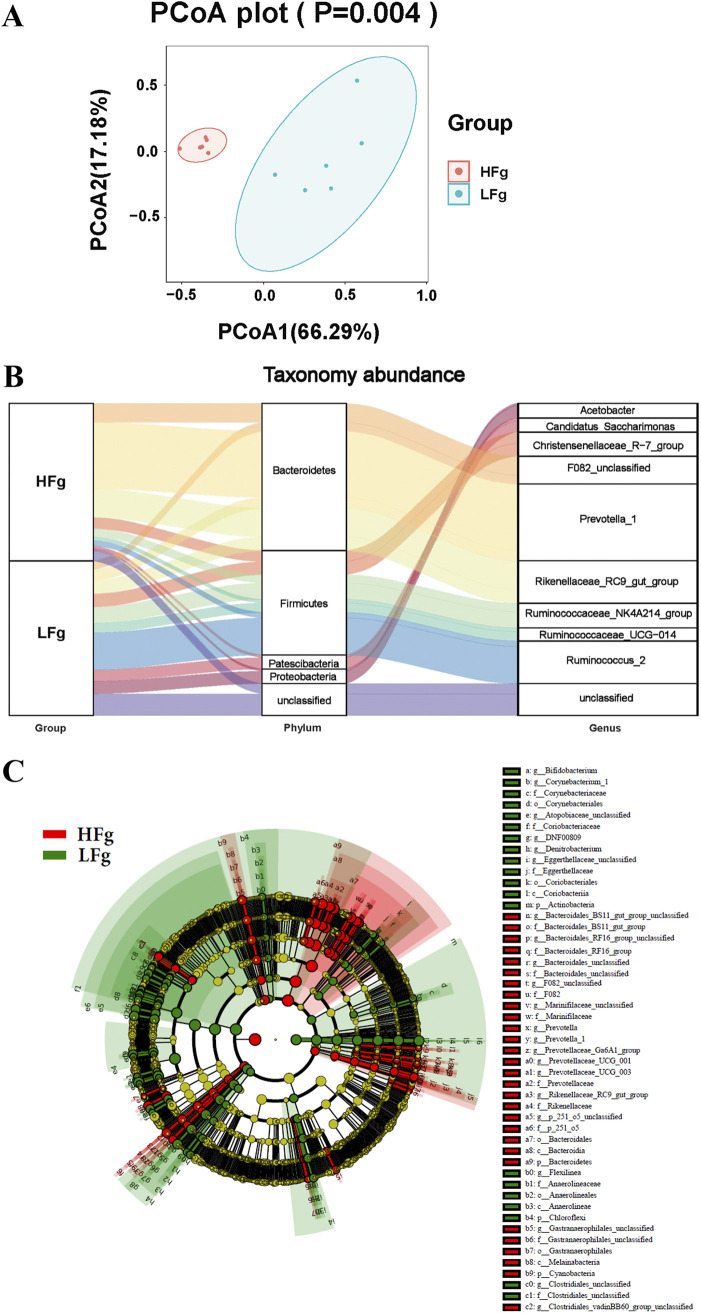
Effect of high- and low-fiber diets on ruminal microbiota of dairy goats. (A) Principal-coordinate analysis of ruminal microbiota from the groups HFg (goats with high-fiber diets) and LFg (goats with low-fiber diets). The data were statistically analyzed based on ANOSIM. (B) Sankey diagram of the HFg and LFg groups in the dominant bacteria phyla with top 5 abundance and the dominant bacterial genera with top 10 abundance; (C) differential taxonomic abundance between the HFg and LFg groups was analyzed by linear discriminate analysis coupled with effect size measurements (LEfse) as a cladogram. (A linear discriminant analysis [LDA] threshold value of >3 and *P* value of <* *0.05 are shown.)

By feeding high-fiber and low-fiber diets to dairy goats, significant differences in ruminal microbiota were identified using beta diversity analysis (Fig. 2A). Furthermore, significant differences in the top 5 most abundant phyla and the top 10 most dominant genera were identified between the low-fiber (LFg) and high-fiber (HFg) goat groups (Fig. 2B). At the phylum level, the significantly higher relative abundance of *Actinobacteria*, *Planctomycetes*, *Patescibacteria*, *Firmicutes*, and *Chloroflexi*, and significantly lower relative abundance of *Bacteroidetes*, *Cyanobacteria*, *Spirochaetes*, *Lentisphaerae*, and *Kiritimatiellaeota* were identified in the LFg group compared to the HFg group (Fig. S2A and Table S1). At the genus level, a total of 80 differential genera were identified between the two groups. Of these, the top 10 most dominant differential genera included the significantly higher relative abundance of *Ruminococcus_2* and *Candidatus_Saccharimonas* and significantly lower relative abundance of *Prevotella_1*, *Rikenellaceae_RC9_gut_group*, and *F082_unclassified* in the LFg group (Fig. S2B, Table S2). Furthermore, LEfse (linear discriminant analysis [LDA] effect size) analysis identified a total of 4 phyla, 4 classes, 5 orders, 14 families, and 22 genera between the HFg and LFg groups (least discriminant analysis value [LDA] of >3), which again proved significantly higher-fiber-utilization bacteria in the HFg groups (Fig. 2C).

**FIG 3:**
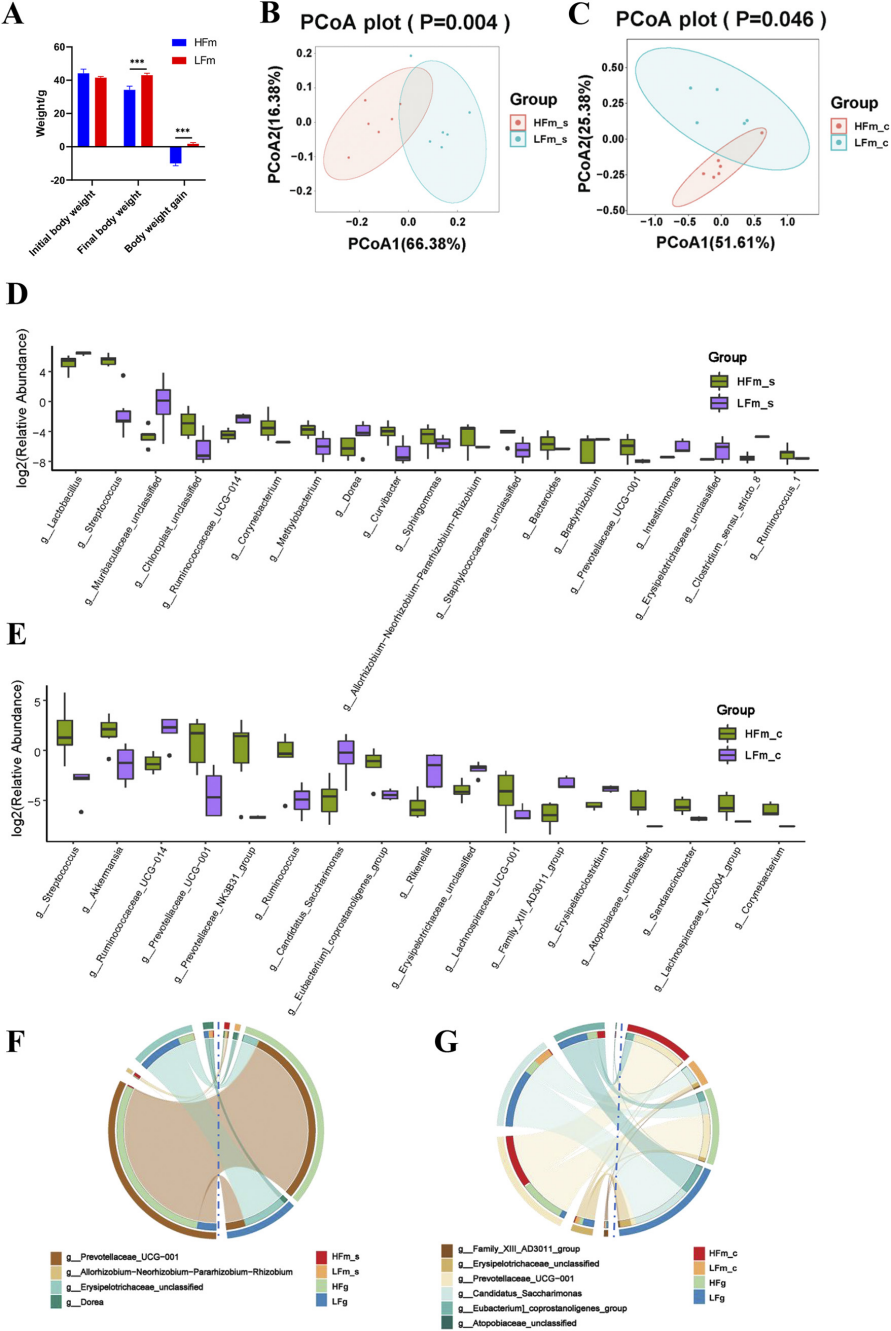
Effect of high- and low-fiber diets on the body weight and intestinal microbiota of mice. (A) Effect of high-fiber diet and low-fiber diet on the weight of mice. The data were analyzed using Student's *t* test and are expressed as the means with standard errors. (B and C) Principal-coordinate analysis (PCoA) of the small intestinal bacterial community between the groups HFm (high-fiber-fed mice) and LFm (low-fiber-fed mice) (B) and the colonic bacterial community between the HFm and LFm groups (C). The data of panels B and C were statistically analyzed based on ANOSIM. (D and E) Differential bacteria at the genus level (*P* < 0.05) of the small intestine bacterial community (D) and the colonic bacterial community (E) between the HFm and LFm groups. The Mann-Whitney U test was used to identify significantly different bacteria. All bacteria listed here were significantly differential bacteria with *P* values of <0.05 between the two groups. All of the data are expressed as means with standard deviations. (F and G) Shared differential bacteria at the genus level between the rumen microbiota of dairy goats from the HFg and LFg groups and the small intestinal microbiota of mice from the HFm and LFm groups (F) and the rumen microbiota of dairy goats from the HFg and LFg groups and the colonic microbiota of mice from the HFm and LFm group (G).

Unlike the goats, which could utilize the high-fiber diets with high efficiency, the body weight and the body weight gain of mice were significantly lower after they were fed a high-fiber diet (Fig. 3A). We speculated that this difference was induced by the lack of enzymatic digestion of high-fiber diets of mice and the lack of fiber-degrading bacteria in the gastrointestinal tract of mice. We performed 16S rRNA gene sequencing on the small intestine and colon contents of these two groups of mice. A principal-coordinate analysis (PCoA) revealed significant differences in the microbial community structure of the small intestine and colon between mice fed high- versus low-fiber diets (Fig. 3B and C). At the phylum level, compared to the high-fiber diet (HFm) group, the low-fiber diets (LFm) significantly lower the relative abundance of *Cyanobacteria* in the small intestine. Furthermore, a significantly lower relative abundance of *Verrucomicrobia* and significantly higher relative abundance of *Patescibacteria* in the colon of LFm mice were identified, compared to the HFm mice (Fig. S3A and B). Furthermore, the top 10 most dominant genera of the small intestine and colon of mice from the HFm and LFm groups were identified (Fig. S3C and D). Totals of 19 and 17 differential genera in the small intestine and colon of mice, respectively, were identified between HFm and LFm groups (Fig. 3D and E; Table S2). Notably, the significantly lower numbers of differential genera were identified due to high-fiber diet intake in mice, rather than in goats.

Furthermore, we analyzed the common differential genera induced by the high- and low-fiber diets, between the dairy goats and mice. The differences in tendency of four genera, *Prevotellaceae_UCG-001*, *Allorhizobium-Neorhizobium-Pararhizobium-Rhizobium*, *Erysipelotrichaceae_unclassified*, and *Dorea*, that responded to the high-fiber diets, were consistent in the small intestine of mice and the rumen of dairy goats (Fig. 3F). There were 4 of 6 other genera, including the *Family_XIII_AD3011_group*, *Erysipelotrichaceae_unclassified*, *Prevotellaceae_UCG-001*, and *Candidatus_Saccharimonas* consistently differential in the colon of mice and rumen of dairy goats that responded to the high dietary fiber (Fig. 3G). In addition, *Prevotellaceae_UCG-001* was the only genus that consistently differential in the small intestine and colon of mice that responded to the high fiber diets.

### RMT can increase fiber utilization in antibiotic-pretreated mice and may be related to the colonization of cellulolytic bacteria in the small intestine and colon microbes.

**FIG 4:**
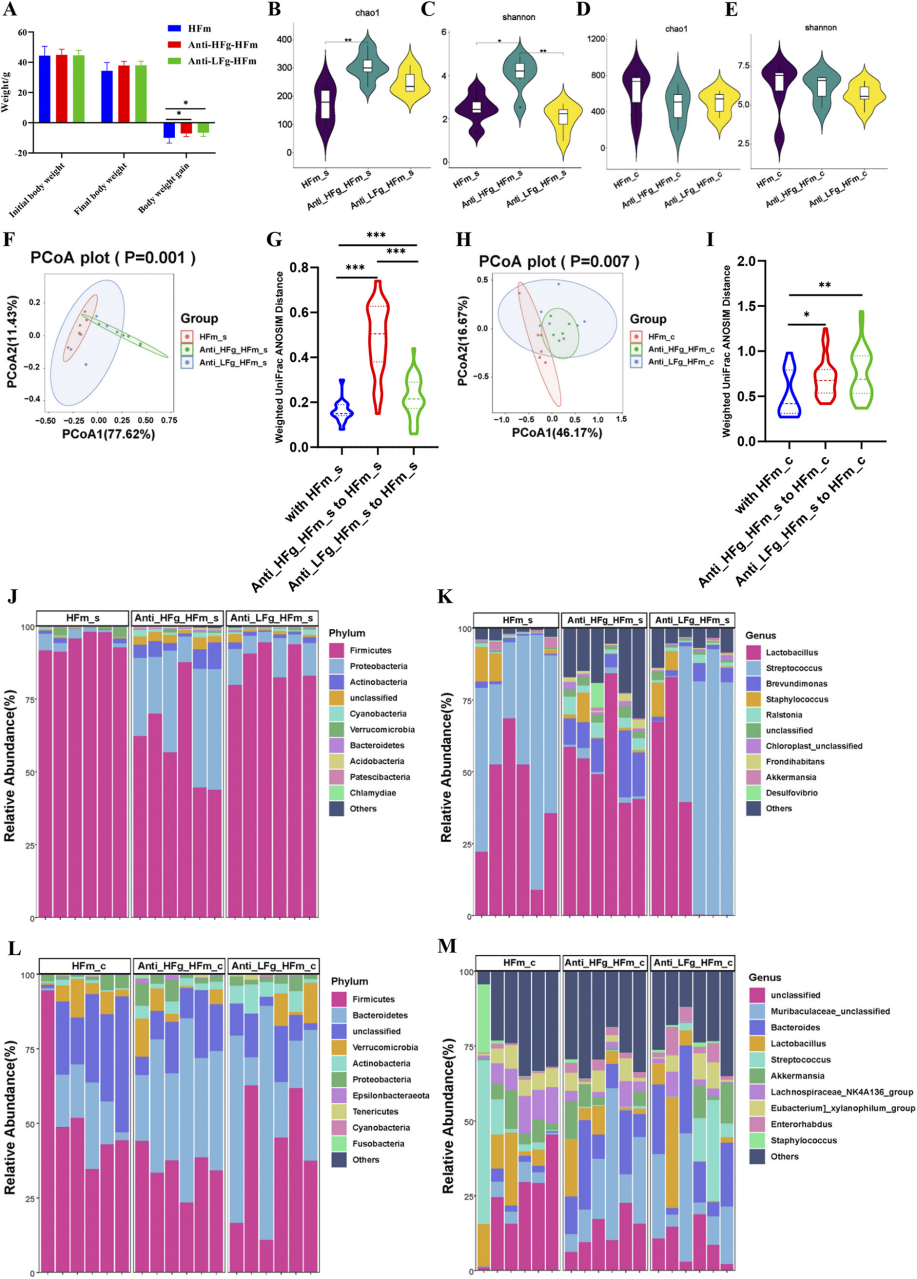
Effects of ruminal microbiota transplant on the significantly changed growth performance and intestinal microbial composition of antibiotic-pretreated mice fed a high-fiber diet compared with the normally fed mice fed a high-fiber diet, but without antibiotics and RMT treatment. (A) Effects of ruminal microbiota transplant on the weight of antibiotic-pretreated mice fed a high-fiber diet compared with the normally fed mice fed a high-fiber diet but without antibiotics and RMT treatment. The data were analyzed using ANOVA. If a significant treatment effect was observed by ANOVA, the significant difference between treatments was identified by Duncan’s multiple-comparison test. All of the data are expressed as means with standard errors. (B and C) Chao1 index (B) and Shannon index (C) of the small intestine bacterial community of mice among the groups HFm (high-fiber-fed mice), Anti-HFg-HFm (antibiotic-pretreated mice that received ruminal microbiota from high-fiber-fed goats and meanwhile were fed high-fiber diets), and Anti-LFg-HFm (antibiotic-pretreated mice that received ruminal microbiota from low-fiber-fed goats and meanwhile were fed high-fiber diets). (D and E) Chao1 index (D) and Shannon index (E) of the colonic bacterial community of mice among the HFm, Anti-HFg-HFm, and Anti-LFg-HFm groups. The data of panels B to E were statistically analyzed using the Kruskal-Wallis test with Dunn's *post hoc* test. (F) Principal-coordinate analysis of the small intestine bacterial community of mice among the HFm, Anti-HFg_HFm, and Anti_LFg_HFm groups; (G) small intestinal microbial weighted UniFrac ANOSIM distances among the HFm, Anti-HFg-HFm, and Anti-LFg-HFm groups; (H) principal-coordinate analysis of the colonic bacterial community of mice among the HFm, Anti-HFg-HFm, and Anti-LFg-HFm groups; (I) colonic microbial weighted UniFrac ANOSIM distances among the HFm, Anti-HFg-HFm, and Anti-LFg-HFm groups. The data of panels F and H were statistically analyzed based on ANOSIM. The data of panels G and I were statistically analyzed using the Kruskal-Wallis test with Dunn's *post hoc* test. (J and K) Differences in the relative abundance of the small intestinal bacterial at the phylum level (J) and genus level (K) among the HFm, Anti-HFg-HFm, and Anti-LFg-HFm groups; (L and M) differences in the relative abundance of the colonic bacterial at the phylum level (L) and genus level (M) among the HFm, Anti-HFg-HFm, and Anti-LFg-HFm groups.

Exploiting the strong fiber utilization of ruminants, we further transplanted rumen microbes to antibiotic-pretreated mice to study their fiber utilization. Compared to the HFm group, significantly higher body weight gain was identified both in the antibiotic-pretreated mice that received ruminal microbes from goats with a high-fiber diet (Anti-HFg-HFm) and goats with a low-fiber diet (Anti-LFg-HFm) (Fig. 4A). Similarly, compared to the antibiotic-treated mice with high-fiber diets (Anti-HFm), significantly higher body weight gain was identified in the Anti-LFg-HFm group (Fig. S4A). We supposed that these results could be induced by the colonization of some fiber-degrading bacteria in the gut of mice after RMT and then improving the fiber utilization ability and growth performance of mice.

Furthermore, we studied intestinal and colonic microbiota differences of mice after RMT. Compared to the HFm group, the diversity and richness (Shannon and Chao1 indices) of the small intestinal microbial communities in the Anti-HFg-HFm group were both significantly higher, while the diversity (Shannon index) of the small intestinal microbial communities was significantly lower in the Anti-LFg-HFm group than in the Anti-HFg-HFm group (Fig. 4B and C). The diversity and richness (Shannon and Chao1 indices) of the colonic microbial communities were not significantly different between the HFm, Anti-HFg-HFm, and Anti-LFg-HFm groups (Fig. 4D and E). Compared to the Anti-HFm group, the diversity and richness (Shannon and Chao1 indices) of the small intestinal microbial communities in the Anti-LFg-HFm group were both significantly lower (Fig. S4B and C). Significantly lower diversity (Shannon index) of the small intestinal microbial communities was identified in the Anti-LFg-HFm group than in the Anti-HFg-HFm group (Fig. S4C). Further, the richness (Chao1 index) of the colonic microbial communities was not significantly different (Fig. S4D). The diversities (Shannon index) of the colonic microbial communities in the Anti-HFg-HFm and Anti-LFg-HFm groups were both significantly higher than those in the Anti-HFm group (Fig. S4E). Further beta diversity analysis identified significant distinctions among the HFm, Anti-HFg-HFm, and Anti-LFg-HFm groups and among the Anti-HFm, Anti-HFg-HFm, and Anti-LFg-HFm groups when the colonic and small intestinal microbial compositions were considered separately (Fig. 4F to I; Fig. S4F to I).

**FIG 5:**
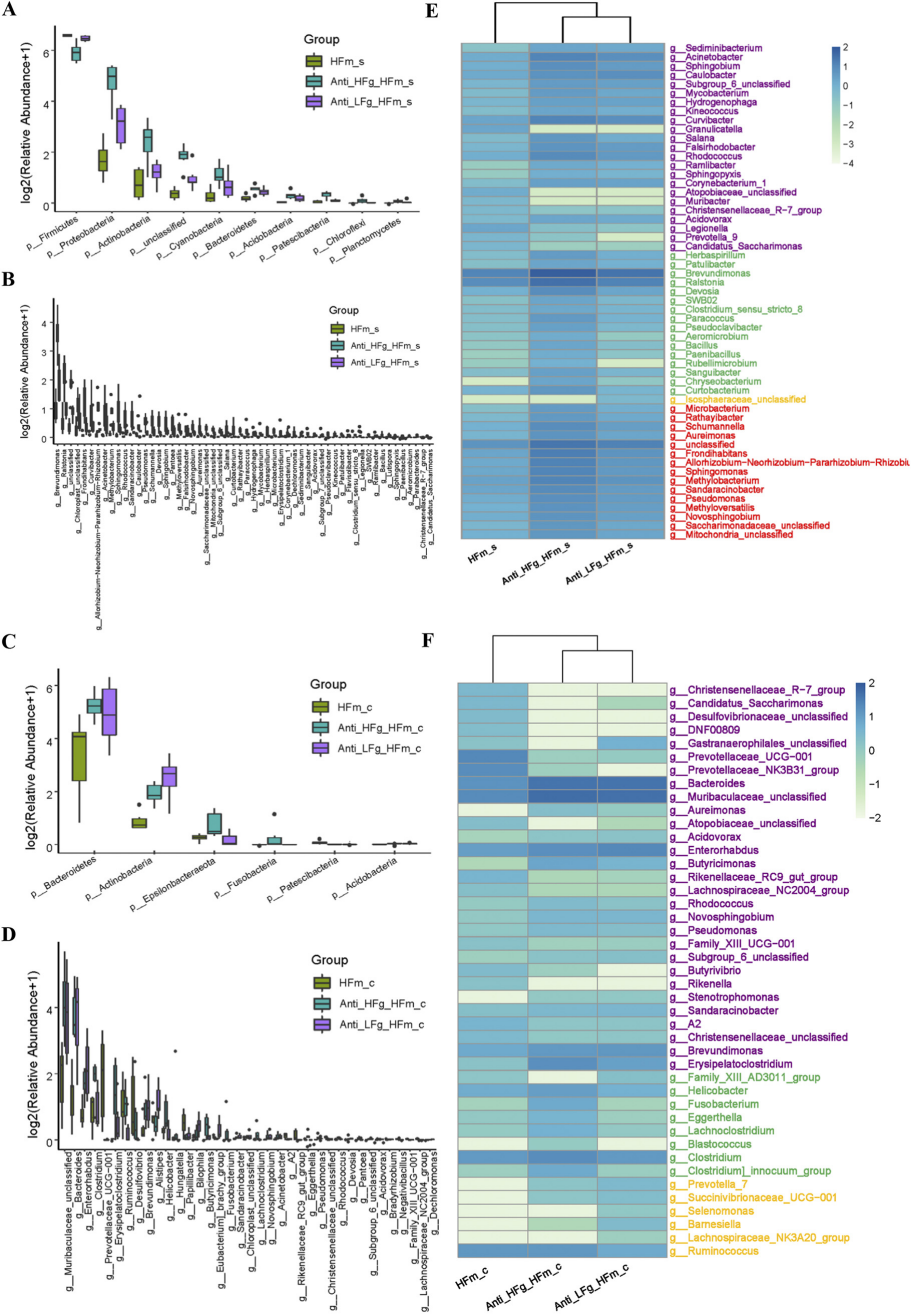
Significantly differential bacteria between antibiotic-treated mice receiving RMT treatment compared with the mice without treatment with antibiotics and RMT when they all received a high-fiber diet. (A and B) Small intestinal differential bacteria at the phylum level (A) and the genus level (B) among the groups HFm (high-fiber-fed mice), Anti-HFg-HFm (antibiotic-pretreated mice that received ruminal microbiota from high-fiber-fed goats and meanwhile were fed high-fiber diets), and Anti-LFg-HFm (antibiotic-pretreated mice that received ruminal microbiota from low-fiber-fed goats and meanwhile were fed high-fiber diets); (C and D) colonic differential bacteria at the phylum level (C) and the genus level (D) among the HFm, Anti-HFg-HFm, and Anti-LFg-HFm groups. The data of panels A to D were statistically analyzed using the Kruskal-Wallis test with Dunn's *post hoc* test. All of the data are expressed as means with standard deviations. (E) Shared differential bacteria in the small intestine of mice among the HFm, Anti-HFg-HFm, and Anti-LFg-HFm groups; (F) shared differential bacteria in the colon of mice among the HFm, Anti-HFg-HFm, and Anti-LFg-HFm groups. The Mann-Whitney U test was used to identify significantly different bacteria. All bacteria listed here were significantly differential bacteria with *P* values of <0.05 between the two groups. All data in the heat map are processed by log_10_ and standardized. In detail, the shared differential bacteria in the comparisons of HFm versus Anti-HFg-HFm and HFm versus Anti-LFg-HFm are marked in purple. The shared differential bacteria in the comparisons HFm versus Anti-HFg-HFm and Anti-HFg-HFm versus Anti-LFg-HFm are marked in green. The shared differential bacteria in the comparisons HFm versus Anti-LFg-HFm and Anti-HFg-HFm versus Anti-LFg-HFm are marked in yellow. The shared differential bacteria in the comparisons HFm_s versus Anti-HFg-HFm and HFm versus Anti-LFg-HFm and Anti-HFg-HFm versus Anti-LFg-HFm are marked in red.

Based on the top 10 dominant microorganisms at the phylum and genus levels identified in the small intestine and colon among the Anti-HFg-HFm, Anti-LFg-HFm, HFm and Anti-HFm groups (Fig. 4J to M; Fig. S4J to M), significantly altered bacteria were identified. Compared to the HFm group, the abundance of *Proteobacteria*, *Actinobacteria*, *Cyanobacteria*, *Bacteroidetes*, *Acidobacteria*, *Chloroflexi*, and *Planctomycetes* at the phylum level in the small intestine was significantly higher in the Anti-HFg-HFm and Anti-LFg-HFm groups, and the abundance of *Firmicutes* in the small intestine was significantly lower in the Anti-HFg-HFm and Anti-LFg-HFm groups (Fig. 5A; Table S1). Furthermore, a total of 56 genera were significantly different in the small intestine among the HFm, Anti-HFg-HFm, and Anti-LFg-HFm groups (Fig. 5B; Table S2). Compared to the HFm group, the abundance of *Bacteroidetes*, *Actinobacteria*, *Fusobacteria*, and *Acidobacteria* at the phylum level in the colon was significantly higher in the Anti-HFg-HFm and Anti-LFg-HFm groups, and the abundance of *Epsilonbacteraeota* was significantly higher in the Anti-HFg-HFm group, but significantly lower in Anti-LFg-HFm groups. Furthermore, the abundance of *Patescibacteria* in the colon was significantly lower in the Anti-HFg-HFm and Anti-LFg-HFm groups compared to the HFm group (Fig. 5C; Table S1). Furthermore, a total of 37 significantly different genera were identified in the colon between the HFm and Anti-HFg-HFm groups or between the Anti-HFm and Anti-LFg-HFm groups (Fig. 5D; Table S2). We analyzed the shared differential bacteria between the different compared groups to identify the key fiber digestion bacteria that help the goats and the mice that received RMT to utilize the dietary fiber (Fig. 5E and F; Fig. S5A to F and Tables S1 and S2). Notably, compared to the HFm or the Anti-HFm groups, significantly higher colonization of cellulolytic bacteria in the colon, such as *Bacteroides*, *Ruminococcus*, *Succinivibrionaceae_UCG-001*, *Prevotella_7*, *Lachnospiraceae_NK3A20_group*, and *Selenomonas*, could be the cause of the higher fiber utilization ability of mice after RMT.

### Prediction of significantly altered microbial functions in preantibiotic-treated mice after RMT.

**FIG 6:**
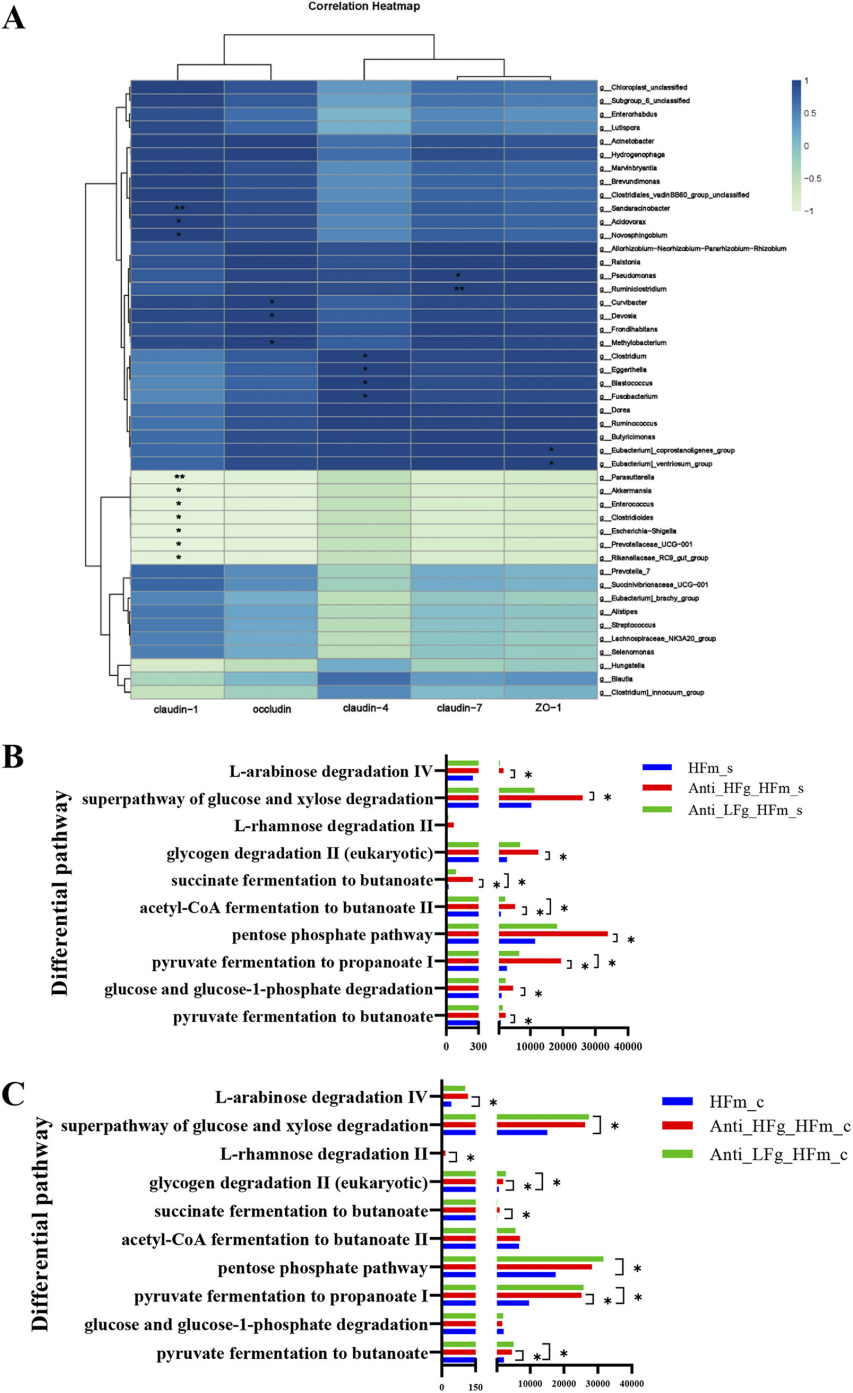
Correlation analysis and function prediction of intestinal and colonic microbiota among the HFm, Anti-HFg-HFm, and Anti-LFg-HFm groups. (A) Pearson correlation between the shared differential bacteria in the colon among the HFm, Anti-HFg-HFm, and Anti-LFg-HFm groups and their relative mRNA expression of tight junction proteins in the colon epithelia. An asterisk indicates the correlation is significant (*P* < 0.05). (B) Prediction of the differential function related to the energy metabolism of the small intestinal microbes among the HFm, Anti-HFg-HFm, and Anti-LFg-HFm groups based on multiple MetaCyc pathways using PICRUSt2; (C) prediction of the differential function related to the energy metabolism of the colonic microbes among the HFm, Anti-HFg-HFm, and Anti-LFg-HFm groups based on multiple MetaCyc pathways using PICRUSt2. The Mann-Whitney U test was used to rank pathways in panels B and C that were significantly differentially changed (*P* < 0.05) in predicted metagenome pathway analysis.

We first analyzed the correlation between the common differential colonic bacteria (Fig. 5E and F) and the colonic epithelial barrier-related indices of mice in the present study. The results show that the abundance of *Sandaracinobacter*, *Acidovorax*, *Novosphingobium*, *Pseudomonas*, *Ruminiclostridium*, *Curvibacter*, *Devosia*, *Clostridium*, *Eggerthella*, *Blastococcus*, *Fusobacterium*, *Eubacterium_coprostanoligenes_group*, and the *Eubacterium_ventriosum_group* were positively correlated with the tight junction protein-related gene expression, indicating that these genera may improve the colonic epithelial barrier (Fig. 6A).

The functional composition profiles of intestinal microorganisms were predicted from 16S rRNA sequencing data using PICRUSt2 (Phylogenetic Investigation of Communities by the Reconstruction of Unobserved States 2). Among the identified differential pathways, we focused on the 10 pathways involved in energy metabolism. After RMT, these energy-related pathways were significantly enhanced in both the small intestine and colon (Fig. 6B and C; Fig. S6A and B). These 10 pathways are also mainly related to butyrate production.

### Effect of low-fiber diet supplementation on the colonization of fiber-degrading bacteria in the intestines after RMT.

**FIG 7:**
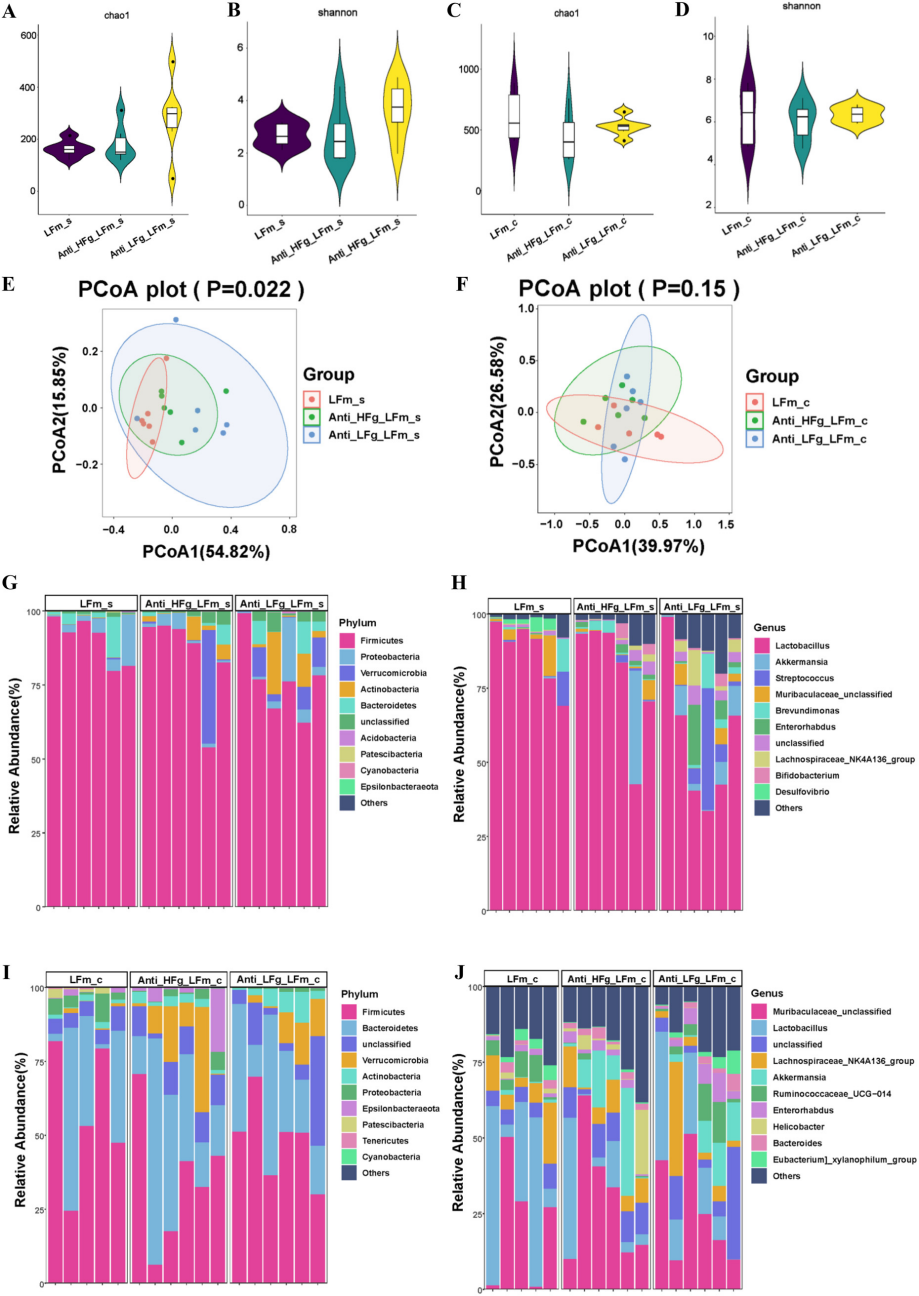
Effects of ruminal microbiota transplant on the significantly changed intestinal microbial composition of antibiotic-pretreated mice fed a low-fiber diet compared with the normally fed mice fed a low-fiber diet, but without antibiotics and RMT treatment. (A and B) Chao1 index (A) and Shannon index (B) of the small intestinal bacterial community of mice among the groups LFm (low-fiber-fed mice), Anti-HFg-LFm (antibiotic-pretreated mice that received ruminal microbiota from high-fiber-fed goats and meanwhile were fed low-fiber diets), and Anti-LFg-LFm (antibiotic-pretreated mice that received ruminal microbiota from low-fiber-fed goats and meanwhile were fed low-fiber diets); (C and D) Chao1 index (C) and Shannon index (D) of the colonic bacterial community of mice among the LFm, Anti-HFg-LFm, and Anti-LFg-LFm groups. The data of panels A to D were statistically analyzed using the Kruskal-Wallis test with Dunn's *post hoc* test. (E) Principal-coordinate analysis of the small intestine bacterial community of mice among the LFm, Anti-HFg-LFm, and Anti-LFg-LFm groups; (F) small intestinal microbial weighted UniFrac ANOSIM distances among the LFm, Anti-HFg-LFm, and Anti-LFg-LFm groups; (G) principal-coordinate analysis of the colonic bacterial community of mice among the LFm, Anti-HFg-LFm, and Anti-LFg-LFm groups; (H) colonic microbial weighted UniFrac ANOSIM distances among the LFm, Anti-HFg_LFm, and Anti-LFg-LFm groups. The data of panels E and G were statistically analyzed based on ANOSIM. The data of panels F and H were statistically analyzed using the Kruskal-Wallis test with Dunn's *post hoc* test. (I and J) Differences in the relative abundance of the colonic bacterial at the phylum level (I) and genus level (J) among the HFm, Anti-HFg-HFm, and Anti-LFg-HFm groups.

**FIG 8:**
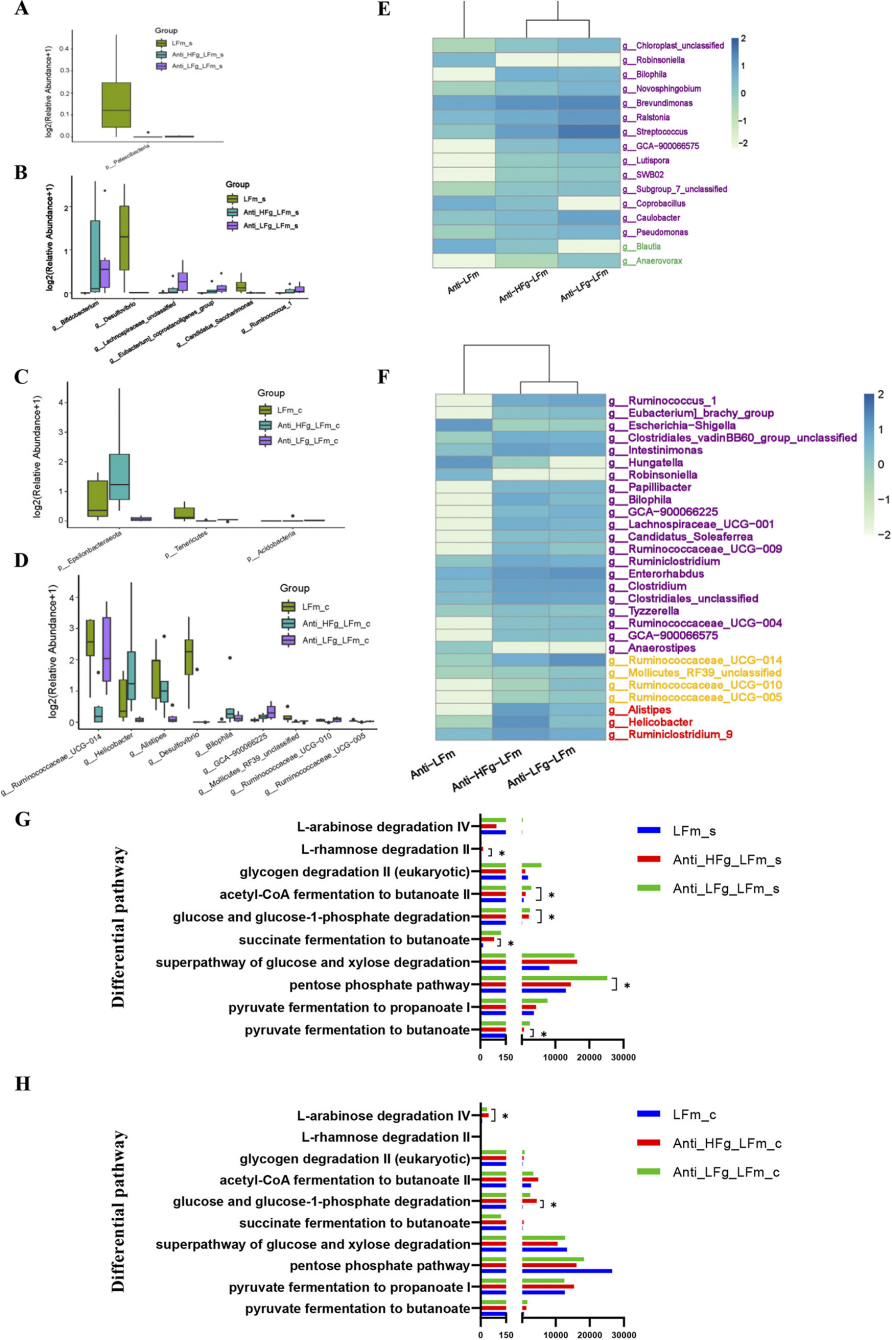
Significantly different bacteria between antibiotic-treated mice receiving RMT treatment compared with the mice without treatment with antibiotics when they all received a low-fiber diet. (A and B) Small intestinal differential bacteria at the phylum level (A) and the genus level (B) among the groups LFm (low-fiber-fed mice), Anti-HFg-LFm (antibiotic-pretreated mice that received ruminal microbiota from high-fiber-fed goats and meanwhile were fed low-fiber diets), and Anti-LFg-LFm (antibiotic-pretreated mice that received ruminal microbiota from low-fiber fed goats and meanwhile were fed low-fiber diets); (C and D) colonic differential bacteria at the phylum level (C) and the genus level(D) among the LFm, Anti-HFg-LFm, and Anti-LFg-LFm groups. The data of panels A to D were statistically analyzed using the Kruskal-Wallis test with Dunn's *post hoc* test. All of the data are expressed as means with standard deviations. (E) Shared differential bacteria in the small intestine of mice among the LFm, Anti-HFg-LFm, and Anti-LFg-LFm groups; (F) shared differential bacteria in the colon of mice among the LFm, Anti-HFg-LFm, and Anti-LFg-LFm groups. The Mann-Whitney U test was used to identify significantly different bacteria. All bacteria listed here were significantly differential bacteria with *P* values of <0.05 between the two groups. All data in the heat map are processed by log_10_ and standardized. In detail, the shared differential bacteria in the comparisons LFm versus Anti-HFg-LFm and LFm versus Anti-LFg-LFm are marked in purple. The shared differential bacteria in the comparisons LFm versus Anti-HFg-LFm and Anti-HFg-LFm versus Anti-LFg-LFm are marked in green. The shared differential bacteria in LFm versus Anti-LFg-LFm and Anti-HFg-LFm versus Anti-LFg-LFm are marked in yellow. The shared differential bacteria in LFm versus Anti-HFg-LFm and LFm versus Anti-LFg-LFm and Anti-HFg-LFm versus Anti-LFg-LFm are marked in red. (G) Prediction of the differential function related to the energy metabolism of the small intestinal microbes among the LFm, Anti-HFg-LFm, and Anti-LFg-LFm groups based on multiple MetaCyc pathways using PICRUSt2; (H) prediction of the differential function related to the energy metabolism of the colonic microbes among the LFm, Anti-HFg-LFm, and Anti-LFg-LFm groups based on multiple MetaCyc pathways using PICRUSt2. The Mann-Whitney U test was used to rank pathways of panels G and H that were significantly different (*P* < 0.05) in predicted metagenome pathway analysis.

Furthermore, we conducted interventions of low-fiber diets and RMT, exploring the differential colonization of ruminal fiber-degrading bacteria affected by the differential fiber. According to alpha diversity analyses, compared to the LFm group, the diversity and richness (Shannon and Chao1 indices) of the small intestinal microbial communities in the Anti-HFg-LFm and Anti-HFg-LFm groups were not significantly different (Fig. 7A to D). Beta diversity analysis identified significant differences in small intestinal microbial composition among the LFm, Anti-HFg-LFm, and Anti-LFg-LFm groups, but no significant differences based on the colonic microbial composition of these 3 groups (Fig. 7E and F). Based on the top 10 dominant microorganisms at the phylum and genus levels in the small intestine among the LFm, Anti-HFg-LFm, and Anti-LFg-LFm groups (Fig. 7G and H), a significantly lower level of small intestinal *Patescibacteria* was identified in Anti-HFg-HFm and Anti-LFg-HFm groups than in the LFm group (Fig. 8A, Table S1). There were 6 significantly different genera identified in the small intestine between the HFm and Anti-HFg-HFm groups and between the Anti-HFm and Anti-LFg-HFm groups (Fig. 8B; Table S2). Similarly, concerning the colonic microbiota (Fig. 7F to I), significantly lower levels of *Tenericutes* and *Acidobacteria* were identified in the Anti-HFg-LFm and Anti-LFg-LFm groups than in the LFm group. Furthermore, the abundance of colonic *Epsilonbacteraeota* was significantly higher in the Anti-HFg-LFm group, but significantly lower in the Anti-LFg-LFm group (Fig. 8C; Table S1). Nine significantly differential genera were identified in the colon of recipient mice between the LFm and Anti-HFg-LFm groups and between the LFm and Anti-LFg-LFm groups (Fig. 8D; Table S2). We analyzed the shared differential bacteria of the small intestine and colon among these 3 groups (Fig. 8E and F) and found that the abundance of less-fiber-degrading bacteria, including *Ruminococcus* and *Lachnospiraceae*, was significantly higher in the intestine, especially in the colon.

The functional composition profiles of intestinal microorganisms were also predicted from the 16S rRNA sequencing data, using PICRUSt2. Similarly, we again focused on the 10 pathways related to energy metabolism and found only 4 and 2 pathways, respectively, significantly changed in the small intestine and colon, and these pathways were not involved in butyric acid production (Fig. 8G and H).

## DISCUSSION

The rumen has a natural cellulose-degrading system and a higher diversity of prokaryotic and eukaryotic microorganisms inside, which play a key role in the effective digestion and utilization of plant ingredients (5–7). In this study, we identified the difference between the ruminal microbiota of ruminants and intestinal (including small intestine and colon) microbiota of monogastric animals and found significantly higher diversity and richness in ruminal microbiota. This result indicated that the unique physiological structure of the rumen may provide a suitable fermentation location for the colonization of more bacteria (5–7). Our results also demonstrated that there were more fiber-degrading bacteria in the rumen, with a higher abundance of *Bacteroides* and a lower abundance of *Firmicutes* (24–25). This result was consistent with previous studies positing that the ratio of *Firmicutes* and *Bacteroides* will change in response to the induction of dietary fiber and consequently improved intestinal health (24–25). It has been reported that polysaccharides from plant-derived fibers, including cellulose, xylan, arabinogalactan, and pectin, and plant-derived starches, including amylose and amylopectin, could act as the main energy source of *Bacteroides* (26–28). This could be the main reason that more *Bacteroides* were identified in the rumen due to the intake of a large amount of fiber in ruminants, and in turn, more *Bacteroides* in ruminants could be determined by the stronger fiber degradation ability than in monogastric animals (29, 30).

By further feeding high- and low-fiber diets to dairy goats, sufficient fiber substrate could also significantly increase ruminal *Bacteroides* abundance. The relative abundance of the genera *Prevotella* and *Bacteroides* was also significantly higher and again proved that more ruminal fiber-degrading bacteria were identified when animals were fed high-fiber diets (31, 32). However, when we fed mice with high- and low-fiber diets, no significant increase in *Bacteroides* was found in either the small intestine or colon. Only some fiber-degrading genera with a low abundance, such as *Prevotella*, *Bacteroides*, and *Ruminococcus*, were higher in the colon of high-fiber-fed mice. These results indicated that the background abundance of fiber digestion bacteria also determines the high-fiber diets’ effects on gut microbiota. Furthermore, the differential colonization of fiber digestion bacteria in the small intestine and colon may be affected by their internal environment, such as pH (33). Notably, the relative abundance of *Akkermansia*, involved in regulating intestinal health, was higher in the colon of mice fed a high-fiber diet, which again proved the potential effects of high levels of fiber on gut health and higher abundance of *Akkermansia* (34, 35).

Then, we gavaged the rumen microbes of dairy goats into mice using the RMT method and found a significantly higher fiber degradation ability in mice, which could be reflected in the significantly higher body weight of mice after RMT when treated with high-fiber diets and the significantly higher relative abundance of fiber-degrading bacteria in the mouse intestine. Furthermore, by comparing the different intestinal bacterial genera in mice with and without RMT, we identified the key fiber-degrading bacterial genera that were significantly higher after transplantation, such as *Ruminococcus* and *Bacteroides*. These fiber-degrading bacteria were colonized mainly in the colon of mice, which was consistent with the intestinal microbiota composition in different intestines of monogastric animals (36). It was also consistent with our previous study finding that the mouse colon can reflect the fermentation characteristics of and has microbiota similar to ruminal microbiota after RMT (20). Furthermore, we analyzed the correlation between the colonic differential microorganisms and the gene expression of tight junction proteins of mice after RMT; the result showed that the fiber digestion bacteria, which positively correlated with the gene expression of tight junction proteins, might be related to the improvement of the colonic barrier. The functional prediction of colonic microbes found that the pathways related to butyrate production in the RMT and high-fiber-treated mice were significantly higher. Here, butyrate, one of the main metabolites of bacterial fermentation in the colon of mice, is the main energy source of intestinal epithelial cells and can directly affect the growth and differentiation of intestinal cells (37–39). In the present study, the levels of *Butyrivibrio* and *Butyricimonas*, suggested as the main butyrate-producing bacteria in previous studies, were significantly higher in RMT and high-fiber-treated mice (28, 40). This suggested these fiber digestion genera may serve as key bacteria to improve colonic barrier function by producing butyrate. In addition, according to the function prediction analysis of colonic microbes, we also found that the mouse colonic microbiota has a stronger glycolytic function, including l-arabinose degradation, glucose and xylose degradation, and l-rhamnose degradation ability after RMT and high-fiber diet treatment, which confirmed that the significantly higher relative weight after transplantation may be induced by the colonization with significantly higher-fiber-degrading bacteria in the intestine of mice (41–43). Overall, these results suggested that these fiber digestion genera could be considered potential probiotics isolated from the rumen for feeding to monogastric animals to improve their fiber digestion and intestinal health.

A further experiment by feeding a low-fiber diet to mice after RMT showed that only a few fiber-degrading bacteria had an increasing trend in the mouse intestine. The significantly lower number and abundance of colonized fiber-degrading bacteria indicated that diet is the main factor determining intestinal microbe colonization (44). Interestingly, *Ruminococcus* serves as the main bacterium degrading complex carbohydrates, presenting higher abundance in both the high-fiber diet and low-fiber diet, indicating its ability to adapt to the complex and dynamic intestinal environment; it could hence serve as an important fiber-digesting bacterium that would be easier to colonize both the rumen of goats the and intestines of mice. Therefore, its functions deserve further study through metagenomic and cultureomic methods (45, 46).

One of the limitations of the present study was that we have not identified these fiber-degrading bacteria at the species level through 16S rRNA gene sequencing, and some unclassified bacterial genera that were significantly higher, which may also participate in fiber digestion, have not been well identified. In future studies, we strongly suggest using metagenomic and cultureomic methods to identify and isolate more fiber-digested probiotics from the rumen (47, 48). Furthermore, these bacteria isolated from rumen can be studied and used to ferment fibrous feed *in vitro*, to improve the digestibility of neutral detergent fiber (NDF), or as a probiotic to improve the degradation of dietary fiber in monogastric animals and even humans, thereby maintaining intestinal health.

In conclusion, the ruminant microbiota was different from gastrointestinal microorganisms of monogastric animals, which contained numerous fiber-degrading bacteria. The RMT experiment from goats to mice has proven that ruminal microbiota could serve as a key factor in utilizing high-fiber diets. Further analysis focused on the comparison of the differential responses of ruminal and colonic microbiota changes to high-fiber diets and the effects of RMT together with the high- and low-fiber diets, respectively, indicated that the background bacteria and dietary fiber content could together determine the colonization of fiber digestion bacteria. Overall, our study provided a new perspective for the development of probiotics with fiber degradation function from the rumen and the importance of use of prebiotics during the intake of probiotics.

## MATERIALS AND METHODS

### Ethics approval statement.

This experiment was conducted at the Animal Research and Technology Center of Northwest A&F University (Yangling, Shanxi, China) and was performed as per the recommended guidelines from the Administration of Affairs Concerning Experimental Animals (Ministry of Science and Technology, China, revised in 2004). The protocol was approved by the Institutional Animal Care and Use Committee of Northwest A&F University.

### Dairy goat feeding and rumen fluid inoculum preparation.

Twelve multiparous ruminally cannulated dairy goats approximately 4 years of age with an average weight of ~50 kg were used in this study. According to their differential diets, dairy goats were randomly assigned to a high-fiber diet group (HFg; *n* = 6), fed a diet containing 70% forage and 30% concentrate mix, and a low-fiber diet group (LFg; *n* = 6), fed a diet with 30% forage and 70% concentrate mix. The goats were housed individually in their tie stalls, with free access to water. A total of 2 kg TMR experimental diet (see Table S3 in the supplemental material) was fed to each goat twice daily at 0800 h and 1700 h. These dairy goats had no history of gastrointestinal diseases or record of antibiotic use within 3 months.

After dairy goats adapted to the diet for 3 weeks, the rumen fluid was collected from all dairy goats at 2 h after the morning feeding for 3 consecutive days for inoculum preparation. The rumen fluid inoculum was performed as per Hu’s reports, with some slight adjustments (20, 49). In brief, fresh rumen fluid was collected 2 h after the morning feeding from dairy goat donors through the rumen fistula, mixed with the rumen fluid of each group of dairy goats, placed inside a sterile and anaerobic collection tube, and then transferred to the laboratory within an hour. The fluid was strained through four layers of sterile cheesecloth and centrifuged at 6,000 × *g* for 15 min. The precipitate without the supernatant was resuspended in 1× phosphate-buffered saline (PBS), and the resulting suspensions were transferred to the recipient mice directly. All of the rumen fluid inoculum preparations were made in an anaerobic incubator at 37°C. Another 50 mL of rumen fluid inoculum was collected and stored at −80°C to analyze the ruminal microbiota.

### Mouse feeding and RMT.

A total of 48 male Kunming (KM) mice weighing 18 to 20 g were obtained from the Laboratory Animal Center of the Fourth Military Medical University and housed in cages in a specific-pathogen-free animal facility at the College of Animal Science and Technology in Northwest A&F University. All of the mice had *ad libitum* access to water and standard chow (which comprised 83.7% carbohydrates, 12.9% protein, and 2.5% fat) for a 10-day adaptation period and were kept under a 12-h/12-h light-dark cycle and at a 25°C temperature during the entire experiment. After the adaptation period, all of the mice were randomly divided into 8 treatment groups: HFm (*n* = 6), LFm (*n* = 6), Anti-HFm (*n* = 6), Anti-LFm (*n* = 6), Anti-HFg-HFm (*n* = 6), Anti-HFg-LFm (*n* = 6), Anti-LFg-HFm (*n* = 6), and Anti-LFg-LFm (*n* = 6). Briefly, the “LFm” in the name of each group indicates the mice were fed a low-fiber (5% cellulose) diet, and “HFm” indicates the mice were fed a high-fiber diet. The “Anti” in the name of each group indicates the mice were treated with antibiotics, and “HFg” or “LFg” means that the mice were inoculated with the ruminal microbiota of the corresponding dairy goat donor from the HFg and LFg groups.

First, the mice in the Anti groups were treated with ampicillin (1 g/L), ciprofloxacin (200 mg/L), and metronidazole (1 g/L), which were dissolved in the drinking water for 3 weeks (50). Then the mice in the Anti groups were infused by intragastric gavage with 0.5 mL of high-concentration antibiotics once a day for 3 days, and those in the HFm and LFm groups were supplied with sterile water. After a 24-h antibiotic-free period, the mice in the Anti-HFg-HFm and Anti-LFg-HFm groups were infused by intragastric gavage with 0.3 mL of mixed rumen fluid derived from dairy goats of the HFg or LFg group for 3 days through the mouth by using a 65-mm straight gavage needle, while the other groups were given equal amounts of 1× PBS.

### Sample collection of mouse recipients.

On the tenth day after RMT, all the mice were weighed and euthanized by exsanguination after the intravenous administration of 10% chloral hydrate solution (100 mg chloral hydrate/kg body weight; Sigma, USA) and immediately dissected. The intestinal contents were collected instantly, as also were those of the duodenum, jejunum, and ileum, and mixed as the small intestine contents, while the contents of the colon were immediately collected and stored directly. All contents were stored in liquid nitrogen for 24 h, and transferred to −80°C until further DNA extraction.

### DNA extraction, PCR amplification, and 16S rRNA gene sequencing.

DNA samples from the rumen fluid of the dairy goats and the small intestinal colonic contents of the mice were extracted using an E.Z.N.A. stool DNA kit (D4015; Omega, Inc., USA) according to the manufacturer’s instructions. Nuclease-free water was used for the blank. The total DNA was eluted in 50 μL of elution buffer and stored in a −80°C freezer until further library preparation and 16S rRNA gene sequencing.

The bacterial hypervariable regions V3 and V4 of the 16S rRNA gene were PCR amplified using bacterial forward and reverse primers 341 (5′-CCTACGGGNGGCWGCAG-3′) and 805 (5′-GACTACHVGGGTATCTAATCC-3′), respectively (51). PCR amplification was performed in a total volume of 25 μL of reaction mixture containing 25 ng of template DNA, 12.5 μL of PCR Premix, 2.5 μL of each primer, and PCR-grade water to adjust the volume. The PCR conditions to amplify the prokaryotic 16S fragments consisted of an initial denaturation at 98°C for 30 s, followed by 32 cycles of denaturation at 98°C for 10 s, annealing at 54°C for 30 s, and extension at 72°C for 45 s, and then a final extension at 72°C for 10 min. The PCR products were confirmed by 2% agarose gel electrophoresis. Throughout the DNA extraction process, ultrapure water was used instead of a sample solution as a negative control, to exclude the possibility of false-positive PCR results. The PCR products were purified with AMPure XT beads (Beckman Coulter Genomics, Danvers, MA, USA) and quantified with Qubit (Invitrogen, USA). After the PCR amplicon library was prepared, its size and quantity were assessed on an Agilent 2100 Bioanalyzer (Agilent, USA) and with the Library quantification kit for Illumina (Kapa Biosciences, Woburn, MA, USA), respectively. The libraries were sequenced on the NovaSeq Miseq platform.

### Illumina sequencing data analysis.

Paired-end reads were assigned to samples based on their unique barcode and truncated by cutting off the barcode and primer sequence. Paired-end reads were merged using FLASH (52). Quality filtering was performed on the raw reads under specific filtering conditions to obtain clean, high-quality tags according to fqtrim (v0.94), and the chimeric sequences were filtered using Vsearch (v2.3.4) (53). After dereplication using DADA2 (54), we obtained a feature table and denoised feature sequences, which are called amplicon sequence variants (ASVs). Here, the detailed indices regarding the sequencing results and quality are listed in Table S4.

Alpha diversity and beta diversity were calculated by normalizing to the same sequences randomly. According to the SILVA (release 132) classifier, the feature abundance was normalized using the relative abundance of each sample (55). The alpha diversity indices of Chao1 and Shannon were applied to analyze the complexity of the species diversity, and the beta diversity of different groups was calculated by QIIME2 (56). BLAST was used for the sequence alignment, and the feature sequences were annotated with the SILVA 138 database for each representative sequence to determine the different taxonomies at the phylum and genus levels.

PICRUSt2 (Phylogenetic Investigation of Communities by the Reconstruction of Unobserved States 2) analysis (https://github.com/picrust/picrust2) (57) was used to predict the metagenome in the samples, the metagenome functions were predicted, and the data were exported into Metacyc database pathways. For 16S rRNA gene sequencing data analysis, all default parameters were used, unless otherwise mentioned.

### Colonic epithelial RNA extraction and quantitative real-time PCR.

The total RNA from colonic epithelial samples from all the mice was extracted using TRIzol reagent (TaKaRa, Beijing, China). Specifically, DNase I was used during the RNA isolation process to avoid contamination with genomic DNA. The quantity and purity of the total RNA were analyzed with a NanoDrop ND-1000 spectrophotometer (Thermo Scientific, MA, USA), and the integrity of the RNA was assessed by gel electrophoresis. Only RNA samples with an optical density at 260/280 nm (OD_260/280_) of >1.8, an OD_260/230_ of >2.0, and good integrity were used for further quantitative real-time PCR (qRT-PCR). Approximately 1 μg of total RNA from the intestinal epithelium was reverse transcribed using the PrimeScript RT reagent kit with gDNA Eraser (TaKaRa, Dalian, China). qRT-PCR was performed using SYBR green PCR master mix (TaKaRa, Dalian, China). A 20-μL PCR mixture was quickly prepared. Primers for β-actin (internal control genes) and the test mRNAs were obtained from our previously published research (20). In brief, the tested mRNAs included genes involved in coding for tight junction proteins (i.e., occludin, claudin-1, claudin-4, claudin-7, and ZO-1). The PCR was conducted in an iCycler iQ5 multicolor real-time PCR detection system (Bio-Rad Laboratories) and programmed as follows: 95°C for 10 min, 40 cycles of 95°C for 10 s, 60°C for 30 s, 72°C for 30 s, and 72°C for 5 min (47). All of the samples were examined in triplicate. All of the data were analyzed using the threshold cycle (2^−ΔΔ^*^CT^*) method (58).

### Statistical analysis.

The statistical evaluation of the growth performance of mice was done by Student's *t* test using SPSS 21.0. After testing the normality and variance homogeneity of the data, the statistical evaluation of the growth performance of mice was analyzed by analysis of variance (ANOVA) using SPSS 21.0. If a significant treatment effect was observed by ANOVA, the significant difference between treatments was identified by Duncan’s multiple-comparison test. All of the data were expressed as means with standard errors. Differences were considered to be statistically significant at *P* values of <0.05.

The taxon abundance for each sample was determined according to the phylum, class, order, family, and genus. The Mann-Whitney U test was performed to compare the levels of microbial alpha diversity between the two compared groups. The Kruskal-Wallis test with Dunn's *post hoc* test was employed to test the microbial alpha diversity differences among the 3 compared groups. The bacterial community was compared for its beta diversity using the distance matrices generated from the principal-coordinate analysis (PCoA) and analysis of similarity (ANOSIM) based on the weighted UniFrac distance. The Mann-Whitney U test was used to rank bacteria that were significantly different (*P* < 0.05) in their genus/species levels and for the predicted metagenome pathway analysis. Correlations between variables were tested by Pearson correlation test and meanwhile visualized by using the corrplot and pheatmap R packages (59).

### Data availability.

All of the data generated or analyzed in this study are included in this article. The sequencing reads have been submitted to the Sequence Read Archive (SRA) of NCBI and are available under project accession no. PRJNA793120.
