# Supplementary material for: Ruminal Microbiota Determines the High-Fiber Utilization of Ruminants: Evidence from the Ruminal Microbiota Transplant
Source: Microbiol Spectr. 2022 Aug 4;10(4):e00446-22. doi: 10.1128/spectrum.00446-22 (PMC9430676; doi:10.1128/spectrum.00446-22)
Supplement: Supplemental file 1 — Supplemental material. Download spectrum.00446-22-s0001.pdf, PDF file, 2.1 MB [file spectrum.00446-22-s0001.pdf]

Ruminal microbiota determined the high fibre utilization of ruminants: evidence from the ruminal microbiota transplant

Xiaodong Chen [a], Fang Yan [a], Tao Liu [a], Yuanling Zhang [a], Xinyi Li [a, b], Mengya Wang [a], Chenguang Zhang [a], Xiurong Xu [a], Lu Deng [a, #], Junhu Yao [a, #], and Shengru Wu [a, #]

[a] College of Animal Science and Technology, Northwest A&F University, Yangling, Shaanxi, China

[b] Department of Medicine, Karolinska Instutet, Solna, Stockholm, Sweden

Running Head: The high fibre utilization of ruminants

[#] Address correspondence to Shengru Wu, Email: wushengru2013@163.com; Junhu Yao, Email: yaojunhu2004@sohu.com; Lu Deng, Email: denglu128128@163.com;

Xiaodong Chen, Fang Yan and Tao Liu contributed equally to this work.

Supplementary Material

FIG S1. Comparison of identified genera between the ruminal microbiota of dairy goats and microbiota of the small intestine and colon in mice. (A) Differences in the relative abundance of bacterial genus level between the rumen microbiota from goats and the small intestinal microbiota and the colonic microbiota from mice.

(B-C) The differential bacteria at the GENUS level ($P < 0.05$) between the rumen microbiota from goats and the small intestinal microbiota from mice (B), and the rumen microbiomes from goats and the colonic microbiomes from mice (C). The Mann-Whitney U test was used to identify significantly different bacteria. All bacteria listed here were significantly differential bacteria with $P < 0.05$ between the two groups.

All the data were expressed as the means with the standard deviation.

FIG S2. The high-fibre diet feeding significantly changed the ruminal bacterial composition of dairy goats when compared to low-fibre diets feeding. The differential bacteria in the rumen microbiota at the phylum level (A) and the genus level (B) between HFg (goats with high fibre diets) and LFg (goats with low fibre diets) groups. The Mann-Whitney U test was used to identify significantly different bacteria. All bacteria listed here were significantly differential bacteria with $P < 0.05$ between the two groups. All the data were expressed as the means with the standard deviation.

FIG S3. Effect of high- and low-fibre diets on the intestinal microbiota of mice.

(A-B) The differential bacteria at the phylum level of the small intestine (A) and of the colon (B) between the HFm (high fibre fed mice) and LFm (low fibre fed mice) groups'

mice. The Mann-Whitney U test was used to identify significantly different bacteria. All bacteria listed here were significantly differential bacteria with $P < 0.05$ between the two groups. All the data were expressed as the means with the standard deviation.

(C-D) Differences in the relative abundance of the small intestinal bacterial genera (C) and the colonic bacterial genera (D)between HFm and LFm groups' mice.

FIG S4. Effects of ruminal microbiota transplant on the significantly changed growth performance and intestinal microbial composition of antibiotics pre-treated mice that fed with high fibre diet when compared with the antibiotics pr-treated mice fed with high fibre diet but without RMT treatment.

(A) Effects of ruminal microbiota transplant on the weight of antibiotics pre-treated mice fed with high fibre diet when compared with the antibiotics pr-treated mice fed with high fibre diet but without RMT treatment. The data were analyzed using the ANOVA test. If a significant treatment effect was observed by ANOVA, the significant difference between treatments was identified by Duncan's multiple comparisons test. All the data were expressed as the means with the standard error.

(B-C) The Chao 1 index (B) and the Shannon index (C) of the small intestine bacterial community of mice among the Anti_HFm (antibiotics pre-treated mice fed with high fibre diets only) group, the Anti_HFg_HFm (antibiotics pre-treated mice that receive ruminal microbiota from high fibre fed goats and meanwhile fed with high fibre diets) group, and Anti_LFg_HFm (antibiotics pre-treated mice that receive ruminal microbiota from low fibre fed goats and meanwhile fed with high fibre diets) group.

(D-E) The Chao 1 index (D) and the Shannon index (E) of the colonic bacterial community of mice among the Anti_HFm group, the Anti_HFg_HFm group, and the Anti_LFg_HFm group. The data of (B-E) were statistically analyzed using the Kruskal-Wallis test with Dunn's post-hoc test.

(F) Principal Coordinates Analysis (PCoA) of the small intestine bacterial community of mice among the Anti_HFm group, the Anti_HFg_HFm, and Anti_LFg_HFm groups.

(G) The small intestinal microbial weighted UniFrac ANOSIM distances among the Anti_HFm group, the Anti_HFg_HFm group, and the Anti_LFg_HFm group.

(H) Principal Coordinates Analysis (PCoA) of the colonic bacterial community of mice among the Anti_HFm group, the Anti_HFg_HFm group, and the Anti_LFg_HFm group.

(I) The colonic microbial weighted UniFrac ANOSIM distances among the Anti_HFm group, the Anti_HFg_HFm group, and the Anti_LFg_HFm group. The data of (F) and (H) were statistically analyzed based on ANOSIM analysis. The data of (G) and (I) were statistically analyzed using the Kruskal-Wallis test with Dunn's post-hoc test.

(J-K) Differences in the relative abundance of the small intestinal bacterial at the phylum level (J) and genus level (K) among the Anti_HFm group, the Anti_HFg_HFm group, and the Anti_LFg_HFm group.

(L-M) Differences in the relative abundance of the colonic bacterial at the phylum level (L) and genus level (M) among the Anti_HFm group, the Anti_HFg_HFm group, and the Anti_LFg_HFm group.

FIG S5. Significantly differential bacteria between antibiotic-treated mice

receiving RMT treatment when compared with the antibiotic-treated mice without RMY when they all received a high fibre diet.

(A-B) The small intestinal differential bacteria at the phylum level (A) and the genus level (B) among Anti_HFm (antibiotics pre-treated mice fed with high fibre diets only) group, the Anti_HFg_HFm (antibiotics pre-treated mice that receive ruminal microbiota from high fibre fed goats and meanwhile fed with high fibre diets) group, and Anti_LFg_HFm (antibiotics pre-treated mice that receive ruminal microbiota from low fibre fed goats and meanwhile fed with high fibre diets) group.

(C-D) The colonic differential bacteria at the phylum level (C) and the genus level(D) among the Anti_HFm group, the Anti_HFg_HFm group, and the Anti_LFg_HFm group. The data of (A-D) were statistically analyzed using the Kruskal-Wallis test with Dunn's post-hoc test. All the data were expressed as the means with the standard deviation.

(E) The shared differential bacteria in the small intestine of mice among the Anti_HFm group, the Anti_HFg_HFm group, and the Anti_LFg_HFm group.

(F) The shared differential bacteria in the colon of mice among the Anti_HFm group, the Anti_HFg_HFm group, and the Anti_LFg_HFm group. The Mann-Whitney U test was used to identify significantly different bacteria. All bacteria listed here were significantly different bacteria with $P < 0.05$ between the two groups. All data in the heat map are processed by log10 and standardized.

The shared differential bacteria in "Anti_HFm vs Anti_HFg_HFm" and "Anti_HFm vs Anti_LFg_HFm" were marked purple.

The shared differential bacteria in "Anti_HFm vs Anti_HFg_HFm" and

“Anti_HFg_HFm vs Anti_LFg_HFm” were marked green.

The shared differential bacteria in “Anti_HFm vs Anti_LFg_HFm” and “Anti_HFg_HFm vs Anti_LFg_HFm” were marked yellow.

FIG S6. Function prediction of intestinal and colonic microbiota among the Anti_HFm group, the Anti_HFg_HFm group, and the Anti_LFg_HFm group.

(A) Prediction of the differential function related to energy metabolism of the small intestinal microbes among Anti_HFm group, the Anti_HFg_HFm group, and the Anti_LFg_HFm group based on multiple MetaCyc pathways using PICRUSt2.

(B) Prediction of the differential function related to the energy metabolism of the colonic microbes among the Anti_HFm group, the Anti_HFg_HFm group, and Anti_LFg_HFm group based on multiple MetaCyc pathways using PICRUSt2. The Mann-Whitney U test was used to rank pathways in (A) and (B) that were significantly differentially ($P < 0.05$) changed in predicted metagenome pathways analysis.

Supplementary Table S1. Differential bacteria at the phylum level in the comparison group appear in this paper.

Supplementary Table S2. Differential bacteria at the genus level in the comparison group appear in this paper.

Supplementary Table S3. Ingredients and nutrient composition of the HFg and LFg groups on a dry matter (DM) basis.

Supplementary Table S4. The statistics of obtained sequences data of 16S RNA gene sequencing in the present study.

Supplementary Material

A

B

C

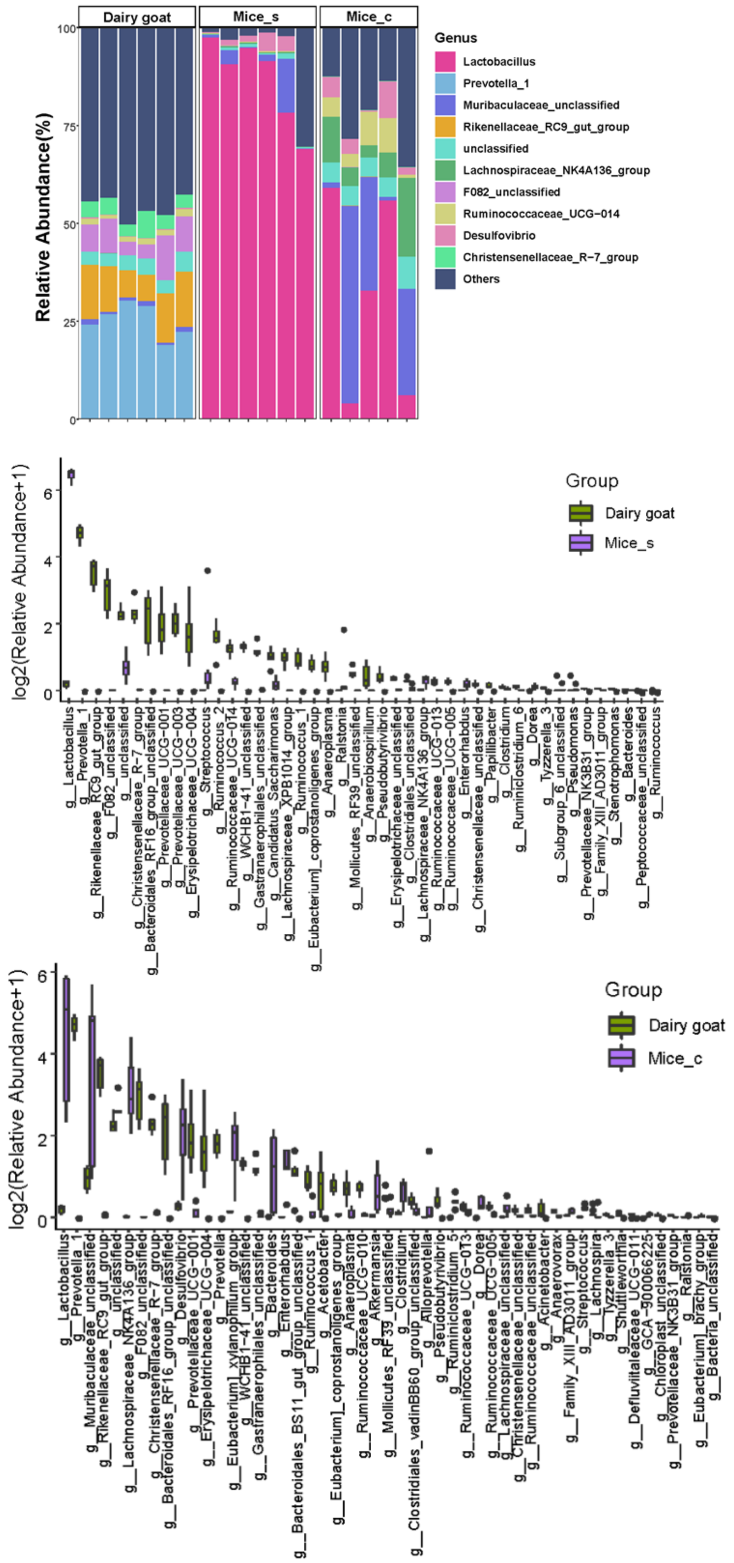

FIG S1. Comparision of identified genera between the ruminal microbiota of dairy goats and microbiota of the small intestine and colon in mice.

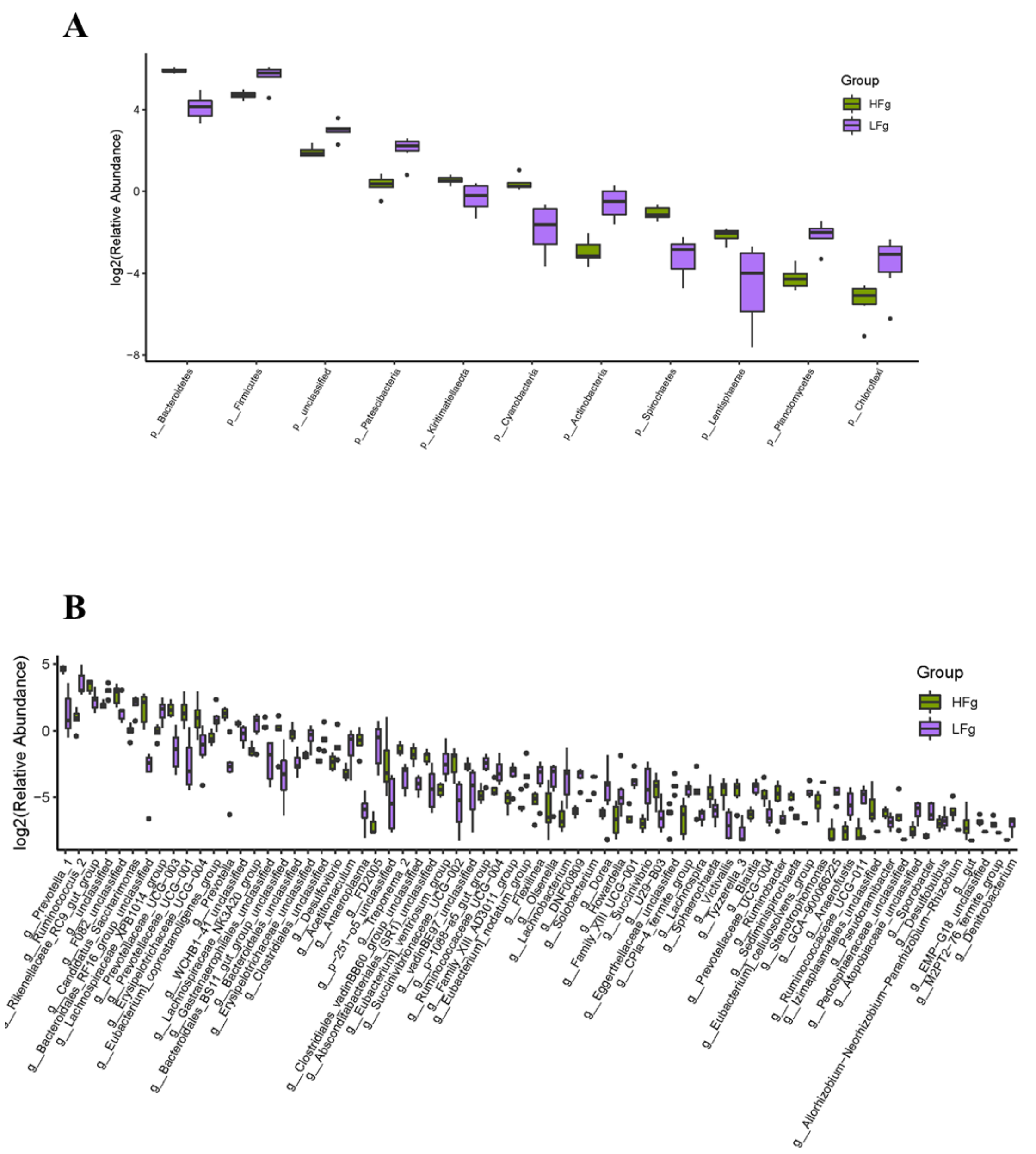

FIG S2. The high-fibre diets feeding significantly changed ruminal bacterial composition of dairy goats when compared low-fibre diets feeding.

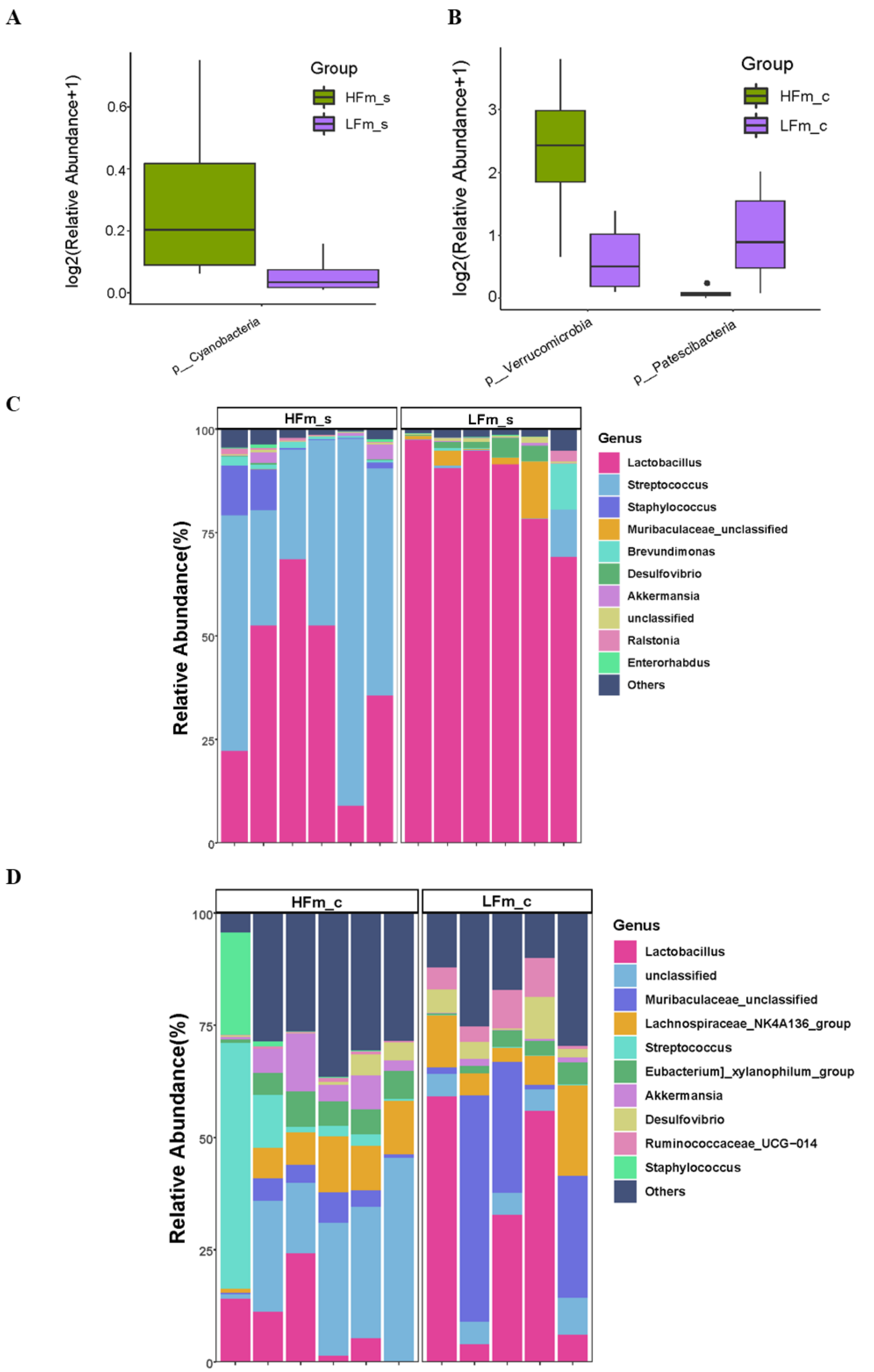

FIG S3. Effect of high- and low-fibre diets on the intestinal microbiota of mice.

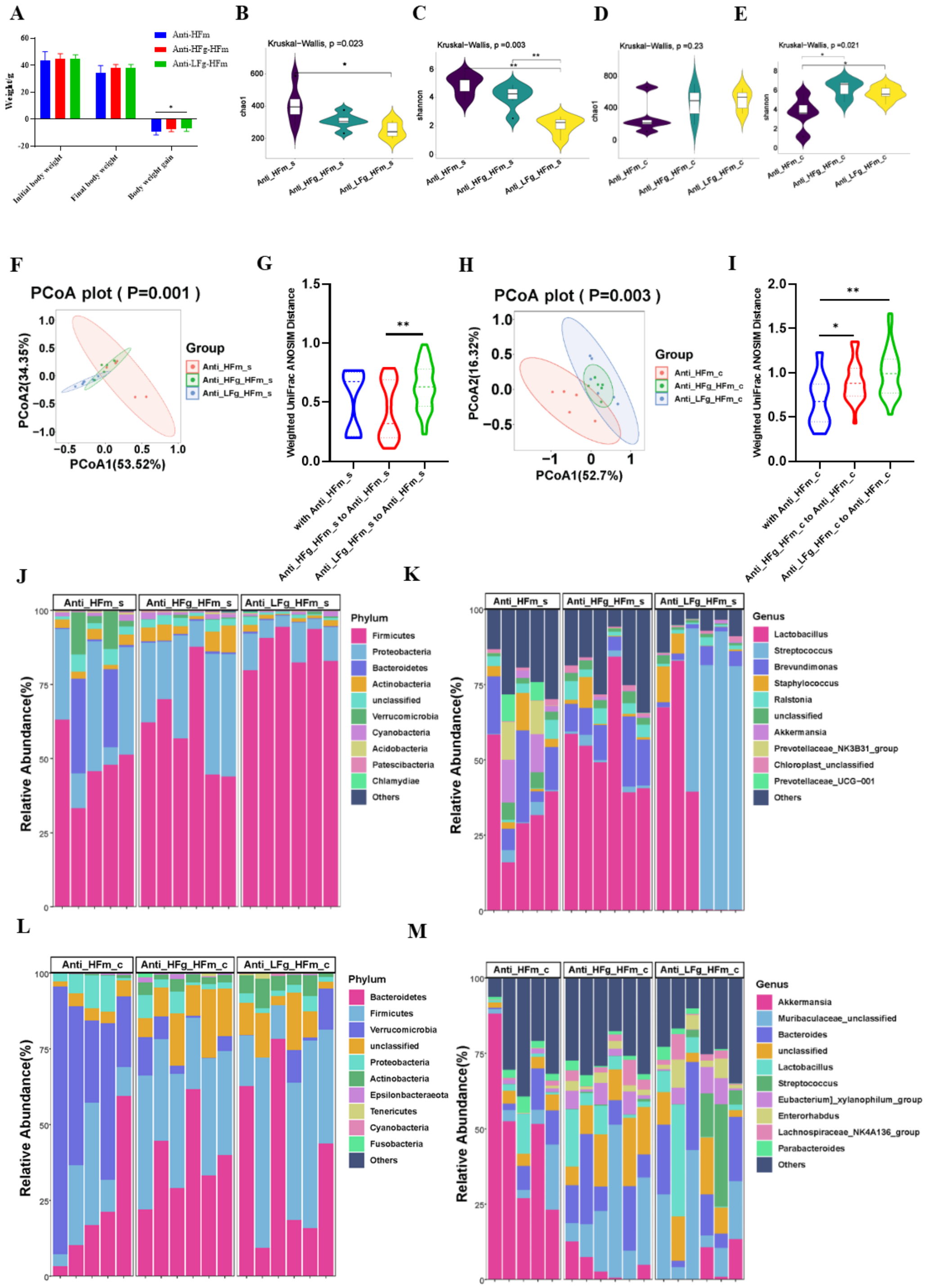

FIG S4. Effects of ruminal microbiota transplant on the significantly changed growth performance and intestinal microbial composition of antibiotics pre-treated mice that fed with high fibre diet when compared with the antibiotics pr-treated mice fed with high fibre diet but without RMT treatment.

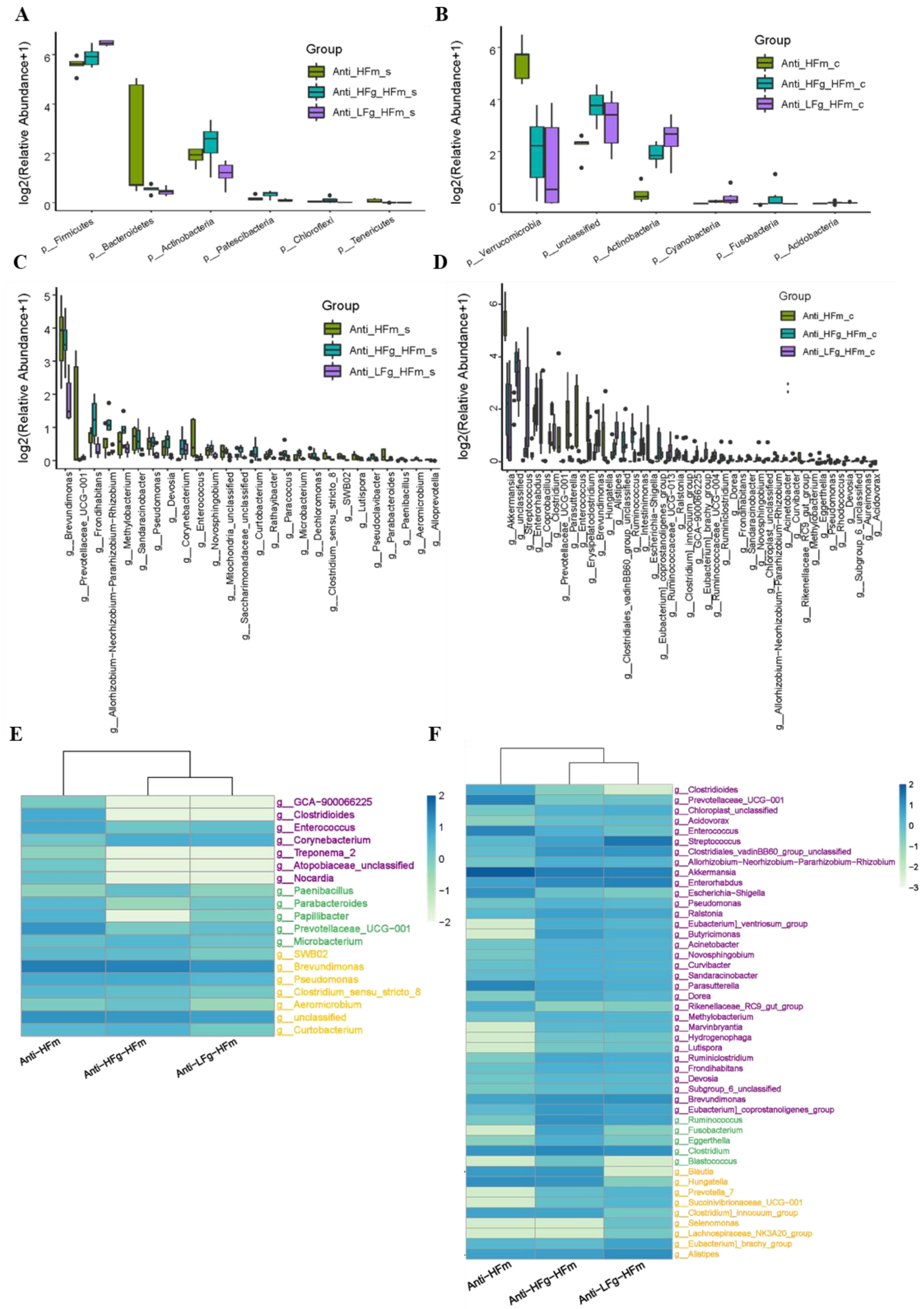

FIG S5. Sighificantly changed bacteria between antibiotic-treated mice receiving RMT treatment when compared with the antibiotic-treated mice without RMY when they all received a high fibre diet.

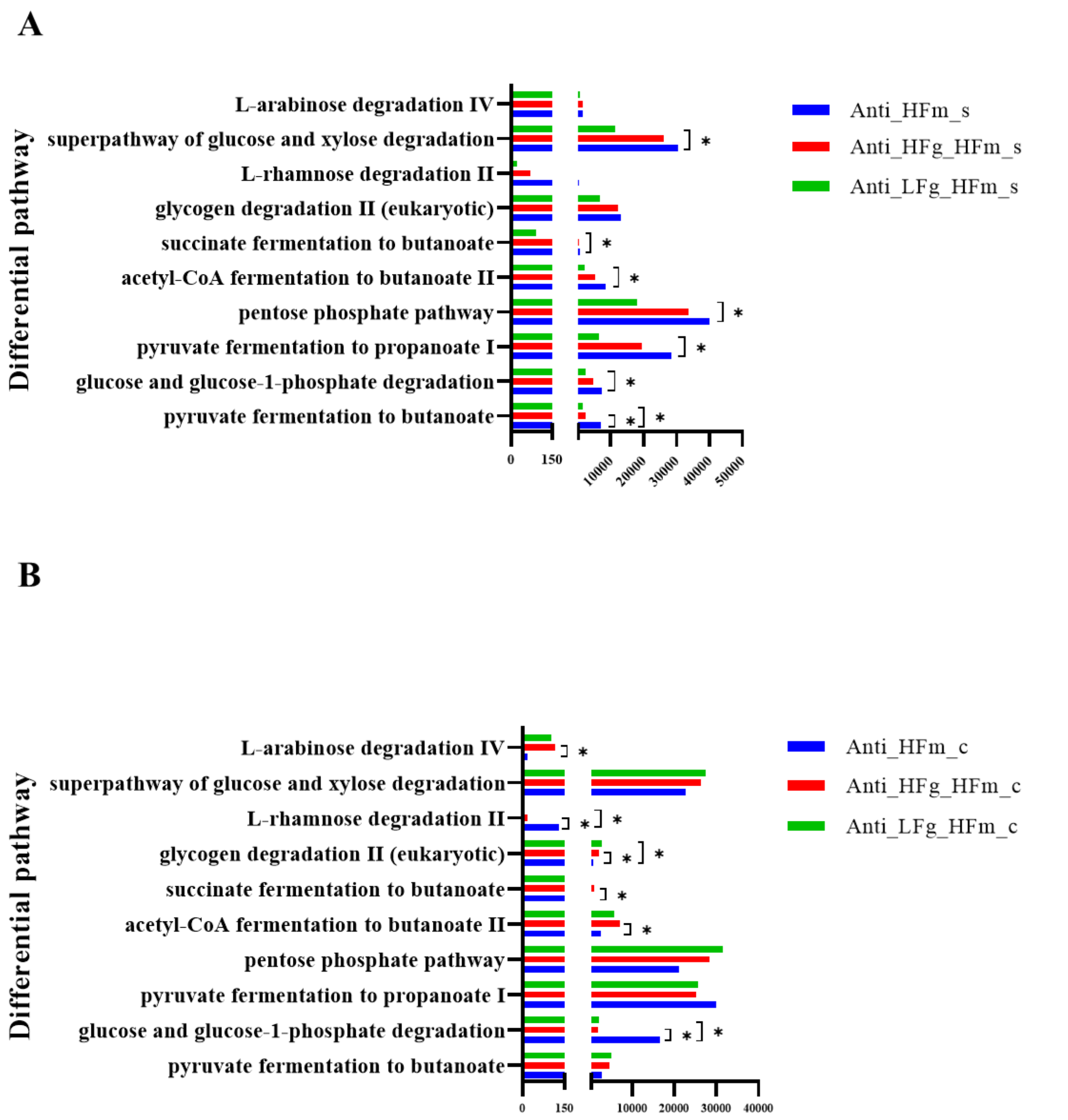

FIG S6. Function prediction of intestinal and colonic microbiota among Anti_HFm group, the Anti_HFg_HFm group, and Anti_LFg_HFm group.

Table S1 Differential bacteria at the phylum level in the comparison group appearing in this paper

Differential bacteria at the phylum level	Relative abundance Differential bacteria		*P* value
	mean_Dairy goat	mean_Mice_s	
p__Kiritimatiellaeota	1.47	0.00	0.00
p__Tenericutes	1.19	0.00	0.00
p__Planctomycetes	0.06	0.00	0.00
p__Firmicutes	26.46	90.26	0.00
p__Bacteroidetes	60.34	3.36	0.00
p__unclassified	3.87	0.67	0.00
p__Patescibacteria	1.30	0.13	0.00
p__Cyanobacteria	1.35	0.03	0.00
p__Chloroflexi	0.03	0.00	0.01
p__Acidobacteria	0.00	0.06	0.05
p__Epsilonbacteraeota	0.00	0.01	0.05
	mean_Dairy goat	mean_Mice_c	
p__Epsilonbacteraeota	0.00	0.82	0.00
p__Kiritimatiellaeota	1.47	0.00	0.01
p__Cyanobacteria	1.35	0.03	0.01
p__Tenericutes	1.19	0.21	0.01
p__Actinobacteria	0.14	1.44	0.01
p__Verrucomicrobia	0.06	0.66	0.02
p__Bacteria_unclassified	0.01	0.00	0.02
p__Bacteroidetes	60.34	28.17	0.04
p__Firmicutes	26.46	57.35	0.04
p__unclassified	3.87	5.62	0.04
	mean_HFg	mean_LFg	

p__Bacteroidetes	60.34	18.50		0.00
p__Cyanobacteria	1.35	0.36		0.00
p__Actinobacteria	0.14	0.75		0.00
p__Spirochaetes	0.49	0.13		0.00
p__Planctomycetes	0.06	0.24		0.00
p__unclassified	3.87	8.24		0.01
p__Patescibacteria	1.30	4.42		0.01
p__Lentisphaerae	0.23	0.07		0.01
p__Kiritimatiellaeota	1.47	0.89		0.01
p__Firmicutes	26.46	52.05		0.04
p__Chloroflexi	0.03	0.11		0.04
	mean_HFm_s	mean_LFm_s		
p__Cyanobacteria	0.23	0.03		0.04
	mean_HFm_c	mean_LFm_c		
p__Verrucomicrobia	5.38	0.66		0.02
p__Patescibacteria	0.07	1.26		0.03
	mean_HFm_s	mean_Anti_HFg_HFm_s	mean_Anti_LFg_HFm_s	
p__Proteobacteria	2.58	28.66	8.35	0.00
p__Firmicutes	94.65	60.93	87.41	0.00
p__Acidobacteria	0.02	0.26	0.14	0.01
p__Patescibacteria	0.03	0.26	0.07	0.01
p__Bacteroidetes	0.15	0.49	0.37	0.01
p__Cyanobacteria	0.23	1.23	0.69	0.01
p__Actinobacteria	0.81	4.93	1.36	0.02
p__Chloroflexi	0.00	0.09	0.01	0.02
p__Planctomycetes	0.00	0.05	0.04	0.02

p__Spirochaetes	0.00	0	0	0.03
	mean_HFm_c	mean_Anti_HFg_HFm_c	mean_Anti_LFg_HFm_c	
p__Fusobacteria	0.00	0.27	0.00	0.00
p__Actinobacteria	0.90	2.89	5.32	0.00
p__Patescibacteria	0.07	0.01	0.01	0.01
p__Acidobacteria	0.01	0.03	0.03	0.01
p__Epsilonbacteraeota	0.20	0.76	0.15	0.02
p__Bacteroidetes	13.71	38.48	38.13	0.03
	mean_LFm_s	mean_Anti_HFg_LFm_s	mean_Anti_LFg_LFm_s	
p__Patescibacteria	0.13	0.00	0.00	0.01
	mean_LFm_c	mean_Anti_HFg_LFm_c	mean_Anti_LFg_LFm_c	
p__Epsilonbacteraeota	0.82	4.94	0.05	0.01
p__Tenericutes	0.21	0.01	0.03	0.03
p__Acidobacteria	0.00	0.03	0.02	0.03
	mean_Anti_HFm_s	mean_Anti_HFg_HFm_s	mean_Anti_LFg_HFm_s	
p__Firmicutes	0.01	48.36	60.93	87.41
p__Tenericutes	0.02	0.06	0.01	0.01
p__Actinobacteria	0.02	2.75	4.93	1.36
p__Patescibacteria	0.04	0.15	0.26	0.07
p__Chloroflexi	0.05	0.03	0.09	0.01
p__Bacteroidetes	0.05	11.99	0.49	0.37
	mean_Anti_HFm_c	mean_Anti_HFg_HFm_c	mean_Anti_LFg_HFm_c	
p__Fusobacteria	0.00	0.27	0.00	0.00
p__Actinobacteria	0.36	2.89	5.32	0.00
p__Verrucomicrobia	48.56	4.76	4.24	0.01
p__Cyanobacteria	0.01	0.07	0.22	0.02

p__Acidobacteria	0.00	0.03	0.03	0.02
p__unclassified	3.83	13.60	9.72	0.04

Table S2 Differential bacteria at the genus level in the comparison group appearing in this paper

Differential bacteria at the genus level	Relative abundance Differential bacteria		*P*
	mean_Dairy goat	mean_Mice_s	value
g__Prevotella_1	25.16	0.00	0.00
g__Bacteroidales_RF16_group_unclassified	4.05	0.00	0.00
g__Prevotellaceae_UCG-003	3.23	0.00	0.00
g__Erysipelotrichaceae_UCG-004	2.76	0.00	0.00
g__WCHB1-41_unclassified	1.47	0.00	0.00
g__Ruminococcus_1	0.89	0.00	0.00
g__Eubacterium]_coprostanoligenes_group	0.72	0.00	0.00
g__Anaeroplasma	0.66	0.00	0.00
g__Pseudobutyrivibrio	0.33	0.00	0.00
g__Ruminococcaceae_UCG-005	0.19	0.00	0.00
g__Christensenellaceae_unclassified	0.13	0.00	0.00
g__Papillibacter	0.11	0.00	0.00
g__Tyzzerella_3	0.05	0.00	0.00
g__Stenotrophomonas	0.03	0.00	0.00
g__Rikenellaceae_RC9_gut_group	10.97	0.00	0.00
g__Prevotellaceae_UCG-001	3.29	0.00	0.00
g__Ruminococcus_2	2.05	0.00	0.00
g__Lachnospiraceae_XPB1014_group	0.99	0.00	0.00
g__Ralstonia	0.00	0.48	0.00
g__Mollicutes_RF39_unclassified	0.44	0.00	0.00
g__Anaerobiospirillum	0.37	0.00	0.00
g__Ruminiclostridium_6	0.08	0.00	0.00
g__Streptococcus	0.01	2.08	0.00

g__Ruminococcaceae_UCG-013	0.20	0.01	0.00
g__Lactobacillus	0.15	87.00	0.00
g__F082_unclassified	7.20	0.01	0.00
g__unclassified	3.87	0.67	0.00
g__Christensenellaceae_R-7_group	4.21	0.01	0.00
g__Ruminococcaceae_UCG-014	1.39	0.18	0.00
g__Gastranaerophilales_unclassified	1.31	0.01	0.00
g__Candidatus_Saccharimonas	1.02	0.13	0.00
g__Erysipelotrichaceae_unclassified	0.28	0.01	0.00
g__Family_XIII_AD3011_group	0.03	0.00	0.00
g__Enterorhabdus	0.00	0.14	0.01
g__Prevotellaceae_NK3B31_group	0.03	0.00	0.01
g__Dorea	0.00	0.07	0.01
g__Lachnospiraceae_NK4A136_group	0.01	0.23	0.01
g__Clostridiales_unclassified	0.23	0.05	0.02
g__Peptococcaceae_unclassified	0.02	0.00	0.02
g__Clostridium	0.08	0.03	0.04
g__Bacteroides	0.02	0.00	0.05
g__Ruminococcus	0.02	0.00	0.05
g__Subgroup_6_unclassified	0.00	0.04	0.05
g__Pseudomonas	0.00	0.04	0.05
	mean_Dairy goat	mean_Mice_c	
g__Prevotella_1	25.16	0.00	0.01
g__Bacteroidales_RF16_group_unclassified	4.05	0.00	0.01
g__Erysipelotrichaceae_UCG-004	2.76	0.00	0.01
g__Prevotella	2.53	0.00	0.01

g__WCHB1-41_unclassified	1.47	0.00	0.01
g__Enterorhabdus	0.00	1.42	0.01
g__Bacteroidales_BS11_gut_group_unclassified	1.14	0.00	0.01
g__Pseudobutyrivibrio	0.33	0.00	0.01
g__Dorea	0.00	0.28	0.01
g__Shuttleworthia	0.05	0.00	0.01
g__Defluviitaleaceae_UCG-011	0.05	0.00	0.01
g__Chloroplast_unclassified	0.04	0.00	0.01
g__Eubacterium]_brachy_group	0.00	0.03	0.01
g__Lachnospiraceae_NK4A136_group	0.01	9.23	0.01
g__F082_unclassified	7.20	0.00	0.01
g__Rikenellaceae_RC9_gut_group	10.97	0.02	0.01
g__Anaerovorax	0.11	0.01	0.01
g__Lactobacillus	0.15	31.63	0.01
g__Christensenellaceae_R-7_group	4.21	0.07	0.01
g__Desulfovibrio	0.21	4.11	0.01
g__Prevotellaceae_UCG-001	3.29	0.10	0.01
g__Eubacterium]_xylanophilum_group	0.09	2.76	0.01
g__Gastranaerophilales_unclassified	1.31	0.03	0.01
g__Ruminococcus_1	0.89	0.12	0.01
g__Eubacterium]_coprostanoligenes_group	0.72	0.04	0.01
g__Ruminococcaceae_UCG-010	0.64	0.04	0.01
g__Akkermansia	0.03	0.66	0.01
g__Ruminiclostridium_5	0.01	0.33	0.01
g__Ruminococcaceae_UCG-005	0.19	0.04	0.01
g__Ruminococcaceae_unclassified	0.13	0.02	0.01

g__Family_XIII_AD3011_group	0.03	0.11	0.01
g__Alloprevotella	0.00	0.47	0.01
g__Anaeroplasma	0.66	0.07	0.01
g__Streptococcus	0.01	0.14	0.01
g__Lachnospira	0.06	0.01	0.01
g__Tyzzerella_3	0.05	0.01	0.01
g__Christensenellaceae_unclassified	0.13	0.02	0.01
g__Acetobacter	0.80	0.00	0.02
g__Prevotellaceae_NK3B31_group	0.03	0.00	0.02
g__Bacteroides	0.02	1.55	0.02
g__Clostridium	0.08	0.58	0.02
g__Bacteria_unclassified	0.01	0.00	0.02
g__Clostridiales_vadinBB60_group_unclassified	0.32	0.12	0.03
g__Acinetobacter	0.14	0.00	0.04
g__Ralstonia	0.00	0.03	0.04
g__Muribaculaceae_unclassified	0.96	21.80	0.04
g__unclassified	3.87	5.62	0.04
g__Mollicutes_RF39_unclassified	0.44	0.15	0.04
g__Ruminococcaceae_UCG-013	0.20	0.09	0.04
g__Lachnospiraceae_unclassified	0.01	0.18	0.05
g__GCA-900066225	0.00	0.05	0.05
	mean_HFg	mean_LFg	
g__Solobacterium	0.00	0.08	0.00
g__Pedosphaeraceae_unclassified	0.02	0.00	0.00
g__Prevotella_1	25.16	4.05	0.00
g__Ruminococcus_2	2.05	14.13	0.00

g__Candidatus_Saccharimonas	1.02	4.33	0.00
g__Bacteroidales_RF16_group_unclassified	4.05	0.14	0.00
g__Prevotellaceae_UCG-003	3.23	0.53	0.00
g__Prevotella	2.53	0.26	0.00
g__Gastranaerophilales_unclassified	1.31	0.31	0.00
g__Bacteroidales_unclassified	0.91	0.21	0.00
g__Anaeroplasma	0.66	0.01	0.00
g__Treponema_2	0.39	0.11	0.00
g__Clostridiales_vadinBB60_group_unclassified	0.32	0.07	0.00
g__Eubacterium]_ventriosum_group	0.05	0.26	0.00
g__Eubacterium]_nodatum_group	0.02	0.12	0.00
g__DNF00809	0.02	0.10	0.00
g__CPla-4_termite_group	0.01	0.05	0.00
g__Sediminispirochaeta	0.03	0.01	0.00
g__Anaerofustis	0.00	0.03	0.00
g__Tyzzerella_3	0.05	0.00	0.01
g__Prevotellaceae_UCG-004	0.04	0.01	0.01
g__Succinivibrionaceae_UCG-002	0.23	0.02	0.01
g__Victivallis	0.05	0.01	0.01
g__unclassified	3.87	8.24	0.01
g__Prevotellaceae_UCG-001	3.29	0.35	0.01
g__Eubacterium]_coprostanoligenes_group	0.72	2.19	0.01
g__Clostridiales_unclassified	0.23	0.75	0.01
g__p-1088-a5_gut_group	0.04	0.19	0.01
g__Dorea	0.00	0.08	0.01
g__Pseudoramibacter	0.01	0.00	0.01

g__Lachnospira	0.06	0.01	0.01
g__Rikenellaceae_RC9_gut_group	10.97	5.21	0.01
g__Lachnospiraceae_XPB1014_group	0.99	3.15	0.01
g__WCHB1-41_unclassified	1.47	0.89	0.01
g__Erysipelotrichaceae_unclassified	0.28	0.81	0.01
g__Absconditabacteriales_(SR1)_unclassified	0.27	0.07	0.01
g__Ruminococcaceae_UCG-004	0.04	0.14	0.01
g__Eubacterium]_cellulosolvens_group	0.00	0.04	0.01
g__Izimaplasmatales_unclassified	0.02	0.00	0.01
g__Howardella	0.01	0.07	0.02
g__Sphaerochaeta	0.05	0.01	0.02
g__F082_unclassified	7.20	3.34	0.02
g__Erysipelotrichaceae_UCG-004	2.76	0.58	0.02
g__Bacteroidales_BS11_gut_group_unclassified	1.14	0.19	0.02
g__Desulfovibrio	0.21	0.55	0.02
g__vadinBE97_unclassified	0.17	0.07	0.02
g__Olsenella	0.02	0.10	0.02
g__U29-B03	0.06	0.01	0.02
g__Blautia	0.00	0.05	0.02
g__Sporobacter	0.00	0.01	0.02
g__Ruminobacter	0.04	0.01	0.02
g__Lachnospiraceae_NK3A20_group	0.38	1.54	0.03
g__Family_XIII_AD3011_group	0.03	0.12	0.03
g__FD2005	0.00	0.62	0.03
g__GCA-900066225	0.00	0.03	0.03
g__Succinivibrio	0.00	0.07	0.03

g__Desulfobulbus	0.00	0.01	0.03
g__Family_XIII_UCG-001	0.01	0.07	0.04
g__p-251-o5_unclassified	0.47	0.03	0.04
g__Acetitomaculum	0.11	0.57	0.04
g__Flexilinea	0.03	0.11	0.04
g__Lachnobacterium	0.01	0.12	0.04
g__Ruminococcaceae_UCG-011	0.00	0.03	0.04
g__Atopobiaceae_unclassified	0.00	0.01	0.04
g__Eggerthellaceae_unclassified	0.01	0.05	0.05
g__Stenotrophomonas	0.03	0.01	0.05
g__Allorhizobium-Neorhizobium-Pararhizobium-Rhizobium	0.01	0.00	0.05
g__gut	0.01	0.00	0.05
g__EMP-G18_unclassified	0.01	0.00	0.05
g__M2PT2-76_termite_group	0.01	0.00	0.05
g__Denitrobacterium	0.00	0.01	0.05
	mean_HFm_s	mean_LFm_s	
g__Corynebacterium	0.17	0.00	0.00
g__Lactobacillus	40.14	87.00	0.00
g__Streptococcus	49.94	2.08	0.00
g__Prevotellaceae_UCG-001	0.02	0.00	0.01
g__Curvibacter	0.08	0.01	0.01
g__Intestinimonas	0.00	0.01	0.01
g__Methylobacterium	0.09	0.01	0.01
g__Chloroplast_unclassified	0.23	0.02	0.02
g__Allorhizobium-Neorhizobium-Pararhizobium-Rhizobium	0.05	0.00	0.02
g__Erysipelotrichaceae_unclassified	0.00	0.01	0.02

g__Sphingomonas	0.05	0.01	0.02
g__Bacteroides	0.03	0.00	0.02
g__Muribaculaceae_unclassified	0.05	3.31	0.03
g__Dorea	0.02	0.07	0.03
g__Ruminococcaceae_UCG-014	0.03	0.18	0.03
g__Staphylococcaceae_unclassified	0.05	0.01	0.03
g__Ruminococcus_1	0.01	0.00	0.03
g__Clostridium_sensu_stricto_8	0.01	0.01	0.05
g__Bradyrhizobium	0.02	0.01	0.05
	mean_HFm_c	mean_LFm_c	
g__Sandaracinobacter	0.02	0.01	0.01
g__Streptococcus	12.17	0.14	0.01
g__Family_XIII_AD3011_group	0.01	0.11	0.01
g__Erysipelatoclostridium	0.02	0.07	0.01
g__Prevotellaceae_NK3B31_group	2.91	0.00	0.01
g__Ruminococcaceae_UCG-014	0.48	5.29	0.01
g__Erysipelotrichaceae_unclassified	0.07	0.29	0.01
g__Rikenella	0.02	0.40	0.02
g__Akkermansia	5.38	0.66	0.02
g__Prevotellaceae_UCG-001	3.91	0.10	0.02
g__Eubacterium]_coprostanoligenes_group	0.54	0.04	0.02
g__Atopobiaceae_unclassified	0.03	0.00	0.02
g__Corynebacterium	0.01	0.00	0.02
g__Ruminococcus	1.27	0.04	0.03
g__Candidatus_Saccharimonas	0.06	1.26	0.03
g__Lachnospiraceae_NC2004_group	0.02	0.00	0.03

g__Lachnospiraceae_UCG-001	0.10	0.01		0.04
	mean_HFm_s	mean_Anti_HFg_HFm_s	mean_Anti_LFg_HFm_s	
g__Rathayibacter	0.01	0.18	0.05	0.00
g__Sandaracinobacter	0.07	0.63	0.15	0.00
g__Allorhizobium-Neorhizobium-Pararhizobium-Rhizobium	0.05	1.16	0.23	0.00
g__Mitochondria_unclassified	0.03	0.21	0.07	0.00
g__Microbacterium	0.00	0.16	0.03	0.00
g__Methylobacterium	0.09	0.97	0.28	0.00
g__Methyloversatilis	0.03	0.32	0.12	0.00
g__Saccharimonadaceae_unclassified	0.02	0.25	0.06	0.00
g__Devosia	0.02	0.50	0.07	0.00
g__unclassified	0.30	2.67	1.10	0.00
g__Novosphingobium	0.03	0.29	0.10	0.00
g__Frondihabitans	0.07	1.54	0.28	0.00
g__Mycobacterium	0.01	0.14	0.06	0.00
g__Clostridium_sensu_stricto_8	0.01	0.06	0.02	0.00
g__Brevundimonas	1.04	12.13	2.96	0.00
g__Ralstonia	0.49	3.81	1.27	0.00
g__Schumannella	0.03	0.48	0.13	0.00
g__Sphingomonas	0.05	1.01	0.21	0.00
g__Pseudomonas	0.04	0.47	0.16	0.00
g__Sediminibacterium	0.00	0.08	0.05	0.00
g__Sphingobium	0.03	0.39	0.15	0.00
g__SWB02	0.00	0.06	0.01	0.00
g__Aureimonas	0.02	0.26	0.07	0.00
g__Aeromicrobium	0.00	0.04	0.00	0.00

g__Caulobacter	0.04	0.34	0.41	0.00
g__Hydrogenophaga	0.01	0.11	0.08	0.00
g__Acinetobacter	0.04	0.91	0.43	0.00
g__Subgroup_6_unclassified	0.02	0.17	0.10	0.00
g__Patulibacter	0.00	0.07	0.02	0.01
g__Curvibacter	0.08	1.00	0.43	0.01
g__Pseudoclavibacter	0.00	0.10	0.01	0.01
g__Herbaspirillum	0.01	0.17	0.03	0.01
g__Rhodococcus	0.03	0.67	0.18	0.01
g__Paracoccus	0.01	0.20	0.02	0.01
g__Salana	0.01	0.22	0.04	0.01
g__Dechloromonas	0.01	0.11	0.02	0.01
g__Kineococcus	0.01	0.07	0.03	0.01
g__Paenibacillus	0.00	0.04	0.00	0.01
g__Pantoea	0.02	0.38	0.14	0.01
g__Bacillus	0.00	0.06	0.01	0.01
g__Chloroplast_unclassified	0.23	1.23	0.67	0.01
g__Falsirhodobacter	0.02	0.29	0.14	0.02
g__Erysipelatoclostridium	0.01	0.16	0.02	0.02
g__Lutispora	0.00	0.05	0.01	0.02
g__Sanguibacter	0.01	0.11	0.01	0.03
g__Flavisolibacter	0.00	0.06	0.02	0.03
g__Christensenellaceae_R-7_group	0.01	0.00	0.00	0.03
g__Ramlibacter	0.00	0.05	0.03	0.03
g__Sphingopyxis	0.00	0.03	0.02	0.03
g__Legionella	0.07	0.00	0.00	0.03

g__Acidovorax	0.01	0.07	0.04	0.04
g__Subgroup_7_unclassified	0.01	0.08	0.03	0.04
g__Parabacteroides	0.00	0.00	0.02	0.04
g__Corynebacterium_1	0.01	0.09	0.08	0.04
g__Curtobacterium	0.02	0.22	0.02	0.04
g__Candidatus_Saccharimonas	0.01	0.00	0.00	0.05
	mean_HFm_c	mean_Anti_HFg_HFm_c	mean_Anti_LFg_HFm_c	
g__Prevotellaceae_UCG-001	3.91	0.00	0.01	0.00
g__Bacteroides	1.86	14.60	15.69	0.00
g__Fusobacterium	0.00	0.27	0.00	0.01
g__Lachnospiraceae_NC2004_group	0.02	0.00	0.00	0.01
g__Enterorhabdus	0.75	2.37	4.24	0.01
g__Subgroup_6_unclassified	0.00	0.02	0.02	0.01
g__Rhodococcus	0.01	0.04	0.02	0.01
g__Muribaculaceae_unclassified	3.41	21.06	17.98	0.01
g__Rikenellaceae_RC9_gut_group	0.10	0.00	0.00	0.02
g__Acidovorax	0.00	0.02	0.02	0.02
g__Desulfovibrio	1.64	0.95	0.01	0.02
g__Eggerthella	0.01	0.09	0.00	0.02
g__Eubacterium]_brachy_group	0.06	0.02	0.25	0.02
g__Helicobacter	0.20	0.76	0.15	0.02
g__Erysipelatoclostridium	0.02	2.14	0.58	0.02
g__Pseudomonas	0.01	0.06	0.03	0.02
g__Butyricimonas	0.00	0.36	0.10	0.02
g__Clostridium	0.94	2.77	1.08	0.02
g__Hungatella	0.01	0.96	0.00	0.03

g__Bradyrhizobium	0.00	0.01	0.01	0.03
g__Family_XIII_UCG-001	0.02	0.00	0.00	0.03
g__Lachnoclostridium	0.01	0.15	0.01	0.03
g__Bilophila	0.06	0.13	0.39	0.03
g__Novosphingobium	0.01	0.07	0.08	0.03
g__Pantoea	0.00	0.03	0.01	0.03
g__Sandaracinobacter	0.02	0.07	0.07	0.03
g__Dechloromonas	0.00	0.01	0.01	0.03
g__Alistipes	0.59	0.41	1.36	0.03
g__Negativibacillus	0.02	0.01	0.00	0.04
g__Brevundimonas	0.29	1.03	1.15	0.04
g__Ruminococcus	1.27	1.21	0.26	0.04
g__A2	0.09	0.01	0.01	0.04
g__Christensenellaceae_unclassified	0.06	0.01	0.01	0.04
g__Devosia	0.01	0.03	0.02	0.05
g__Acinetobacter	0.02	0.07	0.06	0.05
g__Chloroplast_unclassified	0.02	0.06	0.08	0.05
g__Papillibacter	0.46	0.06	0.10	0.05
	mean_LFm_s	mean_Anti_HFg_LFm_s	mean_Anti_LFg_LFm_s	
g__Candidatus_Saccharimonas	0.13	0.00	0.00	0.01
g__Bifidobacterium	0.00	1.44	0.98	0.02
g__Desulfovibrio	1.94	0.01	0.01	0.02
g__Eubacterium]_coprostanoligenes_group	0.00	0.05	0.11	0.03
g__Lachnospiraceae_unclassified	0.01	0.07	0.25	0.04
g__Ruminococcus_1	0.00	0.04	0.06	0.05
	mean_LFm_c	mean_Anti_HFg_LFm_c	mean_Anti_LFg_LFm_c	

g__Desulfovibrio	4.11	0.38	0.01	0.01
g__Alistipes	2.43	1.68	0.12	0.01
g__Helicobacter	0.82	4.94	0.05	0.01
g__Ruminococcaceae_UCG-014	5.29	0.47	5.68	0.01
g__Ruminococcaceae_UCG-010	0.04	0.00	0.06	0.01
g__Ruminococcaceae_UCG-005	0.04	0.00	0.02	0.01
g__Mollicutes_RF39_unclassified	0.15	0.01	0.03	0.03
g__GCA-900066225	0.05	0.12	0.28	0.04
g__Bilophila	0.02	0.68	0.11	0.05
	mean_Anti_HFm_s	mean_Anti_HFg_HFm_s	mean_Anti_LFg_HFm_s	
g__Aeromicrobium	0.01	0.04	0.00	0.00
g__SWB02	0.09	0.06	0.01	0.01
g__Clostridium_sensu_stricto_8	0.13	0.06	0.02	0.01
g__Brevundimonas	14.87	12.13	2.96	0.01
g__Devosia	0.30	0.50	0.07	0.01
g__Corynebacterium	0.02	0.39	0.27	0.02
g__Paenibacillus	0.00	0.04	0.00	0.02
g__Microbacterium	0.06	0.16	0.03	0.02
g__Enterococcus	0.65	0.02	0.05	0.02
g__Pseudoclavibacter	0.03	0.10	0.01	0.02
g__Dechloromonas	0.13	0.11	0.02	0.03
g__Parabacteroides	0.13	0.00	0.02	0.03
g__Sandaracinobacter	0.61	0.63	0.15	0.03
g__Paracoccus	0.06	0.20	0.02	0.03
g__Frondihabitans	0.61	1.54	0.28	0.03
g__Pseudomonas	0.49	0.47	0.16	0.03

g__Methylobacterium	0.46	0.97	0.28	0.03
g__Rathayibacter	0.09	0.18	0.05	0.03
g__Saccharimonadaceae_unclassified	0.13	0.25	0.06	0.04
g__Mitochondria_unclassified	0.21	0.21	0.07	0.04
g__Lutispora	0.10	0.05	0.01	0.04
g__Prevotellaceae_UCG-001	3.02	0.01	0.06	0.04
g__Novosphingobium	0.22	0.29	0.10	0.04
g__Alloprevotella	0.02	0.00	0.00	0.05
g__Allorhizobium-Neorhizobium-Pararhizobium-Rhizobium	0.54	1.16	0.23	0.05
g__Curtobacterium	0.11	0.22	0.02	0.05
	mean_Anti_HFm_c	mean_Anti_HFg_HFm_c	mean_Anti_LFg_HFm_c	
g__Prevotellaceae_UCG-001	3.8594	0.0031	0.0067	0.0038
g__Escherichia-Shigella	1.1851	0.0081	0.0026	0.0042
g__Streptococcus	0.0346	0.3492	9.2731	0.0044
g__Hungatella	1.4019	0.9597	0.0011	0.0051
g__Akkermansia	48.5571	4.7596	4.2369	0.0057
g__Enterorhabdus	0.3028	2.3705	4.2382	0.0057
g__Enterococcus	3.592	0.0734	0.0092	0.0063
g__Chloroplast_unclassified	0.0039	0.0636	0.0827	0.0085
g__Ruminococcus	0.0038	1.212	0.2572	0.0091
g__Clostridiales_vadinBB60_group_unclassified	0.0184	0.7527	0.8628	0.0093
g__Coprobacillus	4.2169	1.7402	0.0054	0.0108
g__Curvibacter	0.0107	0.0739	0.0546	0.0108
g__Acinetobacter	0.0049	0.0743	0.064	0.0122
g__Rhodococcus	0.0061	0.0443	0.0184	0.0122
g__Ruminococcaceae_UCG-013	0.001	0.1467	0.4193	0.0132

g__Allorhizobium-Neorhizobium-Pararhizobium-Rhizobium	0.0048	0.0904	0.0488	0.0135
g__Acidovorax	0.0007	0.0151	0.0164	0.0151
g__Pseudomonas	0.0065	0.0574	0.0295	0.016
g__Dorea	0.0016	0.1873	0.0415	0.0166
g__Eggerthella	0.0006	0.0934	0.0032	0.0168
g__Parasutterella	3.4098	0.2348	0.0979	0.0169
g__Clostridium]_innocuum_group	0.2582	0.2508	0.0036	0.0207
g__Ruminiclostridium	0.0018	0.1898	0.0839	0.0207
g__Ralstonia	0.0304	0.3286	0.188	0.0211
g__Subgroup_6_unclassified	0.0032	0.018	0.0247	0.0228
g__Novosphingobium	0.0038	0.0744	0.0811	0.023
g__Sandaracinobacter	0.0186	0.0706	0.0734	0.0233
g__Alistipes	0.3319	0.4053	1.3616	0.0239
g__Eubacterium]_brachy_group	0.0368	0.0227	0.2455	0.0247
g__Clostridium	0.8927	2.7716	1.0759	0.0249
g__GCA-900066225	0.0019	0.1814	0.2031	0.0253
g__Rikenellaceae_RC9_gut_group	0.1295	0.0006	0.0014	0.0254
g__Ruminococcaceae_UCG-004	0.0045	0.1026	0.1779	0.0283
g__Methylobacterium	0.0077	0.0606	0.042	0.0305
g__Erysipelatoclostridium	0.0156	2.1402	0.5815	0.0322
g__Aureimonas	0.001	0.0301	0.0068	0.0335
g__Devosia	0.0048	0.0331	0.0247	0.034
g__unclassified	3.8251	13.6001	9.7211	0.0353
g__Brevundimonas	0.2263	1.0309	1.1514	0.0384
g__Eubacterium]_coprostanoligenes_group	0.027	0.6291	0.2142	0.0408
g__Frondihabitans	0.0082	0.1005	0.0632	0.0453

g__Intestinimonas	0.0863	0.6416	0.4071	0.0464

Table S3. Ingredients and nutrient composition of the HFg and LFg groups on a dry matter (DM) basis

Item	Treatment	
	HFg	LFg
Ingredient, %		
Corn silage	27.74	28.20
Alfalfa hay	42.26	1.80
Corn	15.00	35.00
Corn germ meal	4.10	9.58
Corn gluten feed	7.50	17.50
Cottonseed meal	1.89	4.39
Calcium phosphate	0.11	0.26
Limestone	0.68	1.59
Salt	0.24	0.56
Vitamin-mineral mix[1]	0.18	0.42
Sodium Bicarbonate	0.30	0.70
Nutrient composition		
DM[2], %	52.16	51.64
NE_L, MJ/kg	6.58	7.58
ADF, %	21.6	12.33
NDF, %	31.3	21.35
CP, %	16.4	10.56
Starch, %	28.4	54.53

[1] Vitamin-mineral mix (per kilogram): 450 mg of nicotinic acid, 600 mg of Mn, 950 mg of Zn, 430 mg of Fe, 650 mg of Cu, 30 mg of Se, 45 mg of I, 20 mg of Co, 800 mg of vitamin E, 45,000 IU of vitamin D, and 120,000 IU of vitamin A.

[2] DM, dry matter; NE_L, net energy of lactation; ADF, acid detergent fibre; NDF, neutral detergent fibre; and CP, crude protein.

Table S4 The statistics of obtained sequences data of 16S RNA gene sequencing in the present study.

Group name	Sample	Raw_Tags	Raw_Bases	Valid_Tags	Valid_Bases	Valid%	Q20%	Q30%	GC%
Mice 16S rRNA gene sequencing data									
HFm_s	HFm_s1	84353	42.18M	79973	34.03M	94.81	97.75	93.26	51.57
	HFm_s2	84561	42.28M	78137	33.23M	92.40	96.92	91.19	51.35
	HFm_s3	87493	43.75M	84014	35.80M	96.02	97.97	93.80	51.12
	HFm_s4	81302	40.65M	78681	33.56M	96.78	98.07	94.06	51.26
	HFm_s5	81368	40.68M	74956	31.97M	92.12	96.71	90.75	51.82
	HFm_s6	83699	41.85M	73299	31.19M	87.57	98.00	93.94	51.65
LFm_s	LFm_s1	87010	43.51M	70965	30.27M	81.56	97.48	92.81	50.87
	LFm_s2	82114	41.06M	70081	29.84M	85.35	97.61	93.17	51.46
	LFm_s3	81836	40.92M	67247	28.66M	82.17	96.53	90.70	50.63
	LFm_s4	87869	43.93M	73447	31.31M	83.59	97.48	92.66	51.23
	LFm_s5	85010	42.51M	69625	29.61M	81.90	97.25	92.24	51.36
	LFm_s6	80700	40.35M	71925	30.47M	89.13	97.46	92.69	51.56
Anti_HFm_s	Anti_HFm_s1	84254	42.13M	81663	34.28M	96.92	97.82	93.47	51.66
	Anti_HFm_s2	86487	43.24M	81325	33.89M	94.03	97.80	93.36	53.34
	Anti_HFm_s3	81094	40.55M	78803	32.78M	97.17	98.09	94.11	52.22
	Anti_HFm_s4	80015	40.01M	74956	31.40M	93.68	96.84	90.90	52.99
	Anti_HFm_s5	83872	41.94M	81051	33.88M	96.64	98.08	94.14	52.18
Anti_HFg_HFm_s	Anti_HFg_HFm_s1	81708	40.85M	79596	33.47M	97.42	97.98	93.87	51.93
	Anti_HFg_HFm_s2	81859	40.93M	76008	32.07M	92.85	94.53	85.64	51.96
	Anti_HFg_HFm_s3	86390	43.20M	84174	35.36M	97.43	97.99	93.64	52.44
	Anti_HFg_HFm_s4	84706	42.35M	82220	34.90M	97.07	97.67	92.91	51.18
	Anti_HFg_HFm_s5	84866	42.43M	82608	34.38M	97.34	97.81	93.27	52.36
	Anti_HFg_HFm_s6	86974	43.49M	80506	33.78M	92.56	91.39	79.39	52.56

Anti_LFg_HFm_s	Anti_LFg_HFm_s1	83230	41.62M	78990	33.51M	94.91	94.37	85.11	51.57
	Anti_LFg_HFm_s2	84692	42.35M	82438	35.07M	97.34	97.60	92.61	51.10
	Anti_LFg_HFm_s3	83004	41.50M	80482	34.27M	96.96	97.91	93.53	51.63
	Anti_LFg_HFm_s4	86374	43.19M	83135	35.24M	96.25	95.36	87.60	52.28
	Anti_LFg_HFm_s5	83326	41.66M	81005	34.49M	97.21	97.90	93.67	51.91
	Anti_LFg_HFm_s6	87098	43.55M	84916	36.00M	97.49	97.68	93.18	52.14
HFm_c	HFm_c1	87894	43.95M	81368	34.62M	92.58	97.53	92.84	51.63
	HFm_c2	85886	42.94M	74823	30.87M	87.12	98.00	93.88	53.02
	HFm_c3	82841	41.42M	73530	30.41M	88.76	95.91	88.80	53.12
	HFm_c4	85055	42.53M	74382	30.52M	87.45	98.15	94.26	53.87
	HFm_c5	81940	40.97M	72380	29.72M	88.33	97.95	93.86	54.09
	HFm_c6	83691	41.85M	71682	29.12M	85.65	97.00	91.25	54.26
LFm_c	LFm_c1	84449	42.22M	66980	28.08M	79.31	97.64	93.14	51.94
	LFm_c2	80346	40.17M	69723	29.05M	86.78	97.45	92.68	54.27
	LFm_c3	86923	43.46M	73922	30.91M	85.04	96.23	89.92	52.56
	LFm_c4	81212	40.61M	68741	28.80M	84.64	97.70	93.18	53.11
	LFm_c5	86110	43.05M	72175	29.78M	83.82	97.60	93.04	54.12
Anti_HFm_c	Anti_HFm_c1	83023	41.51M	78972	32.33M	95.12	97.73	93.22	54.11
	Anti_HFm_c2	82098	41.05M	74450	30.77M	90.68	98.01	93.77	53.23
	Anti_HFm_c3	80636	40.32M	71217	29.75M	88.32	97.78	93.29	52.05
	Anti_HFm_c4	82937	41.47M	66681	27.65M	80.40	97.86	93.62	52.89
	Anti_HFm_c5	83714	41.86M	73933	30.78M	88.32	97.87	93.50	53.42
Anti_HFg_HFm_c	Anti_HFg_HFm_c1	86082	43.04M	75145	31.23M	87.29	92.22	81.10	52.29
	Anti_HFg_HFm_c2	84566	42.28M	73837	30.73M	87.31	96.62	90.31	51.23
	Anti_HFg_HFm_c3	80930	40.47M	75444	31.13M	93.22	98.06	94.00	53.32
	Anti_HFg_HFm_c4	81725	40.86M	76690	31.96M	93.84	97.93	93.63	53.89

	Anti_HFg_HFm_c5	82011	41.01M	74779	30.72M	91.18	96.77	90.64	52.29
	Anti_HFg_HFm_c6	82552	41.28M	71382	29.47M	86.47	97.94	93.70	53.56
Anti_LFg_HFm_c	Anti_LFg_HFm_c1	85721	42.86M	77328	32.26M	90.21	97.79	93.39	52.75
	Anti_LFg_HFm_c2	82594	41.30M	70353	29.14M	85.18	97.83	93.58	52.80
	Anti_LFg_HFm_c3	83986	41.99M	70517	29.57M	83.96	97.86	93.48	52.56
	Anti_LFg_HFm_c4	83635	41.82M	76667	31.53M	91.67	96.45	89.86	52.56
	Anti_LFg_HFm_c5	86531	43.27M	73452	30.49M	84.89	97.90	93.68	53.07
	Anti_LFg_HFm_c6	82885	41.44M	76044	31.98M	91.75	97.79	93.38	51.78
Anti_HFg_LFm_s	Anti_HFg_LFm_s1	82415	41.21M	78533	33.43M	95.29	97.48	92.69	51.10
	Anti_HFg_LFm_s2	81356	40.68M	78095	33.25M	95.99	96.96	91.46	50.91
	Anti_HFg_LFm_s3	80727	40.36M	77807	33.12M	96.38	97.31	92.27	50.89
	Anti_HFg_LFm_s4	85189	42.59M	75105	31.89M	88.16	97.74	93.40	52.11
	Anti_HFg_LFm_s5	85538	42.77M	76205	31.71M	89.09	95.87	88.64	52.61
	Anti_HFg_LFm_s6	85217	42.61M	70657	29.82M	82.91	98.00	93.94	53.24
Anti_LFg_LFm_s	Anti_LFg_LFm_s1	81700	40.85M	69870	29.82M	85.52	96.09	89.21	51.20
	Anti_LFg_LFm_s2	85202	42.60M	73758	31.06M	86.57	97.82	93.50	52.10
	Anti_LFg_LFm_s3	85306	42.65M	78250	32.50M	91.73	97.91	93.65	54.56
	Anti_LFg_LFm_s4	84519	42.26M	79103	33.44M	93.59	97.40	92.53	51.73
	Anti_LFg_LFm_s5	85455	42.73M	73106	30.57M	85.55	97.65	93.18	53.00
	Anti_LFg_LFm_s6	86313	43.16M	77489	32.59M	89.78	97.16	91.91	51.91
Anti_HFg_LFm_c	Anti_HFg_LFm_c1	80006	40.00M	67002	27.98M	83.75	94.96	86.91	52.70
	Anti_HFg_LFm_c2	87002	43.50M	77827	32.51M	89.45	97.06	91.51	54.54
	Anti_HFg_LFm_c3	80748	40.37M	69985	29.03M	86.67	97.62	93.03	54.47
	Anti_HFg_LFm_c4	85844	42.92M	71213	29.51M	82.96	97.88	93.65	54.33
	Anti_HFg_LFm_c5	80138	40.07M	70209	28.83M	87.61	97.84	93.56	54.04
	Anti_HFg_LFm_c6	84633	42.32M	75889	31.03M	89.67	92.36	81.73	53.68

Anti_LFg_LFm_c	Anti_LFg_LFm_c1	82645	41.32M	66859	28.15M	80.90	97.55	92.90	53.45
	Anti_LFg_LFm_c2	82911	41.46M	70487	28.88M	85.02	96.45	90.27	53.84
	Anti_LFg_LFm_c3	85887	42.94M	72180	30.29M	84.04	97.89	93.72	54.16
	Anti_LFg_LFm_c4	83720	41.86M	69159	28.58M	82.61	97.74	93.52	53.32
	Anti_LFg_LFm_c5	83385	41.69M	68182	27.97M	81.77	97.75	93.46	53.64
	Anti_LFg_LFm_c6	86689	43.34M	78437	31.93M	90.48	96.62	90.48	53.73
Goat 16S rRNA gene sequencing data									
HFg	HFg1	86406	43.20M	74788	31.16M	86.55	97.75	93.30	53.36
	HFg2	83558	41.78M	72602	30.35M	86.89	97.84	93.54	53.15
	HFg3	83408	41.70M	72281	30.18M	86.66	97.5	92.78	52.79
	HFg4	83675	41.84M	73229	30.49M	87.52	97.89	93.74	53.02
	HFg5	86329	43.16M	76553	31.92M	88.68	97.74	93.35	53.15
	HFg6	81956	40.98M	72689	30.33M	88.69	97.92	93.79	53.39
LFg	LFg1	81971	40.99M	63383	26.09M	77.32	95.59	88.25	52.75
	LFg2	83886	41.94M	68940	28.64M	82.18	96.73	90.58	52.65
	LFg3	84706	42.35M	72798	29.82M	85.94	97.75	93.39	53.02
	LFg4	85030	42.52M	74607	30.73M	87.74	96.38	90.01	52.80
	LFg5	83282	41.64M	73855	30.07M	88.68	97.97	93.96	52.57
	LFg6	84517	42.26M	76892	31.39M	90.98	97.36	92.32	53.08
